# Bacterial Community of Water Yam (*Dioscorea alata* L.) cv. A-19

**DOI:** 10.1264/jsme2.ME21062

**Published:** 2022-05-03

**Authors:** Shunta Kihara, Kosuke Yamamoto, Atsushi Hisatomi, Yuh Shiwa, Chia-Cheng Chu, Kanako Takada, Michel Ouyabe, Babil Pachakkil, Hidehiko Kikuno, Naoto Tanaka, Hironobu Shiwachi

**Affiliations:** 1 Department of International Agricultural Development, Graduate School of International Food and Agricultural Studies, Tokyo University of Agriculture, Tokyo, Japan; 2 Department of Molecular Microbiology, Faculty of Life Sciences, Tokyo University of Agriculture, Tokyo, Japan; 3 Department of Ecological Symbiotic Science, Graduate school of Agriculture, Tokyo University of Agriculture, Tokyo, Japan; 4 NODAI Genome Research Center, Tokyo University of Agriculture, Tokyo, Japan; 5 Miyako Subtropical Training and Research Farm, Tokyo University of Agriculture, Okinawa, Japan

**Keywords:** bacterial community, water yam, orphan crops, 16S rRNA amplicon profiling, fertilization practices

## Abstract

The bacterial community of water yam (*Dioscorea alata* L.) cv. A-19 is vital because it may promote plant growth without the need for fertilization. However, the influence of fertilization practices on the composition and proportion of the bacterial community of water yam cv. A-19 has not yet been extensively examined. Therefore, we herein investigated the diversity and composition of the bacterial community of water yam cv. A-19 cultivated with and without chemical fertilization using amplicon community profiling based on 16S rRNA gene sequences. No significant difference was detected in the growth of plants cultivated with or without chemical fertilization. Alpha diversity indices were significantly dependent on each compartment, and a decrease was observed in indices from the belowground (rhizosphere and root) to aboveground compartments (stem and leaf). The bacterial composition of each compartment was clustered into three groups: bulk soil, rhizosphere and root, and stem and leaf. Chemical fertilization did not significantly influence the diversity or composition of the water yam cv. A-19 bacterial community. It remained robust in plants cultivated with chemical fertilization. The amplicon community profiling of bacterial communities also revealed the dominance of two bacterial clades, the *Allorhizobium-Neorhizobium-Pararhizobium-Rhizobium* clade and *Burkholderia-Caballeronia-Paraburkholderia* clade, with and without chemical fertilization. This is the first study to characterize the bacterial community of water yam cv. A-19 cultivated with and without chemical fertilization.

Agroecosystems are one of the most complex systems, presenting several interconnected components, including plants, microbes, and soil, in natural environments. Interactions between organisms and soil components are vital for maintaining a healthy agroecosystem. Investigations on and the modeling of these complex systems have been conducted to maximize and optimize the effects of these interactions, which will ultimately increase global food production and achieve global food security with the development and sustainability of agroecosystems (Busby *et al.*, 2017; Young and Kinkel, 2017; Toju *et al.*, 2018). Agroecosystems have created a unique food culture for local communities worldwide with variations in staple food products; however, only nine crops (sugarcane, maize, rice, wheat, potatoes, soybean, oil-palm fruit, sugar beet, and cassava) accounted for 66% of all crop production worldwide in 2014. These major crops were selected without the understanding of how important the diversity of crops and food culture is in the world; as a result, this selection process led to the decrease in agrobiodiversity (FAO, 2019).

Yam (*Dioscorea* spp.) is an important tuber crop that is cultivated in tropical and subtropical regions worldwide (Lebot, 2009). In West Africa, yam is crucial as a source of food and for the generation of income (Asiedu and Sartie, 2010), with approximately 67 million tons being produced annually (accounting for approximately 94% of world yam production) (FAOSTAT, 2021). However, despite its importance, yam remains an “orphan crop” (Kennedy, 2003). We recently reported the growth of yams in low fertile alkaline soil on Miyako Island, Okinawa, Japan, which was achieved by the absorption of nitrogen from the air by plant growth-promoting bacteria (PGPB) (Shiwachi *et al.*, 2015; Rezaei *et al.*, 2016, 2017; Takada *et al.*, 2017, 2018; Ouyabe *et al.*, 2019a, 2019b, 2019c, 2020). The involvement of PGPB isolated from yams in nitrogen fixation, phosphate solubilization, indole acetic acid (IAA) production, and siderophore production was demonstrated. We also found that one of the PGPB isolated from water yam (*D. alata* L.) cv. A-19 (hereinafter designated as water yam) affected its growth (Liswadiratanakul *et al.*, 2021), suggesting that yam growth is promoted by PGPB under low fertile conditions.

In West Africa, yam is traditionally cultivated with or without fertilizers after fields recover soil fertility (Asiedu and Sartie, 2010). However, the effects of fertilization on yam growth remain unclear due to discrepancies in previous findings; some studies showed that it increased growth (Hgaza *et al.*, 2012), whereas others did not (Diby *et al.*, 2009). Therefore, many studies have attempted to increase yam productivity by manipulating different soil fertility adaptabilities (Ouyabe *et al.*, 2019a, 2019b). We previously reported the different responses of yam growth and nitrogen absorption from the soil and air among yam varieties under different fertilization practices (Rezaei *et al.*, 2017; Ouyabe *et al.*, 2019a, 2019b). We also found that fertilization practices affected the bacterial community of yam (Ouyabe *et al.*, 2019a). Moreover, bacteria isolated from the endophytic and root compartments differed among yam varieties (Ouyabe *et al.*, 2019a), further corroborating differences in bacterial communities among yam varieties.

In the present study, we used the culture-independent profiling of the bacterial community structure of water yam cultivated with and without chemical fertilization to compare the diversity and composition of their bacterial communities. The primary aims of the present study were to examine the influence of fertilization on the composition of the bacterial community and characterize the optimal endophytic community for yam. Based on 16S rRNA gene amplicon profiling, we characterized the bacterial community and then compared its diversity and community structure under different fertilization practices using culture-independent profiling. Collectively, the present results provide novel insights into the role of the bacterial community on yam growth as well as the responses of yam to fertilization practices.

## Materials and Methods

### Plant material

The present study was conducted between April 24 and August 27, 2018 in a greenhouse at the Tokyo University of Agriculture (Tokyo NODAI), Miyako subtropical farm on Miyako Island, Okinawa, Japan (24°70′N, 125°28′E). Water yam, maintained by the Miyako subtropical farm, was used in this study. On April 27, 20 seed sets were individually planted in a pot (90-L capacity) filled with subsoil collected on Miyako Island. The characteristics of this soil were described by Takada *et al.* (2017). Mean values were as follows: pH 5.1, EC 1.7 μS cm^–1^, and CEC 15.9 me, with 0.06% TN, 0.4% TC, and a C/N ratio of 7.0%. P, K, Ca, and Mg contents were 6.8, 27.5, 74.9, and 32.2‍ ‍mg kg^–1^, respectively.

The experiment was conducted with two treatments: the application of nitrogen at a rate equivalent to 30‍ ‍kg 10 a^–1^ (ten pots) and without nitrogen as a control (ten pots). Nitrogen was applied as urea 60 days after planting (DAP).

### Growth ana­lysis of water yam

Plant growth was measured using physiological parameters. Five plant samples from both treatments were harvested 120 DAP and dried at 80°C for 72 h. Data were collected at 120 DAP. The dry weights of the leaves, stems, and roots were measured.

### Sample preparation for the 16S amplicon sequence ana­lysis

The leaves (Leaf), stems (Stem), roots (R), rhizosphere (Rh), and bulk soil (BS) samples from three plants in both treatments were collected. BS samples were collected from individual pots filled with subsoil without a plant for both treatments. Plants were divided into Leaf, Stem, R, and tuber samples with sterilized scissors. Petioles were removed from leaf samples. Stem samples were cut 1‍ ‍cm above the surface of the pot. R samples were removed 1‍ ‍cm from the stem base. The dead parts of each organ were not included in the present study. Each plant sample was placed in a 1-‍L sterile beaker containing 800‍ ‍mL PBS-S buffer (130‍ ‍mM NaCl, 7‍ ‍mM Na_2_HPO_4_, 3‍ ‍mM NaH_2_PO_4_, pH 7.0, and 0.02% Silwet L-77), and then washed with shaking at 180‍ ‍rpm for 20‍ ‍min (Schlaeppi *et al.*, 2014). A soil suspension (45‍ ‍mL) of R samples from both treatments was transferred to 50-mL sterile tubes and centrifuged at 4,000×*g* for 20‍ ‍min. The pellet generated was defined as the Rh sample (Schlaeppi *et al.*, 2014). R samples were washed with tap water to remove adhered soil and transferred to a new 1-L sterile beaker. Leaf and Stem samples were directly transferred to a new sterile 1-L beaker. The surface sterilization of each plant sample was performed by incubating it in sodium hypochlorite solution (1%) for 5‍ ‍min, followed by 2‍ ‍min in 70% ethanol, and thorough rinsing with sterile water (Carlier and Eberl, 2012). BS, Rh, R, Stem, and Leaf samples were frozen in liquid nitrogen. Frozen samples packed in a styrene foam cold box with dry ice were transported with dry ice to the Tokyo Nodai Setagaya campus and stored at –80°C. Collected samples were used for the extraction of bacterial cells and DNA.

### Bacterial cells and DNA extraction

Bacterial cells were directly extracted from ~50‍ ‍g of Leaf, Stem, and R samples using the bacterial cell enrichment method (Ikeda *et al.*, 2009), which allowed for the elimination of plant organelles and plant genomic DNA. Frozen samples (0.25 g) of BS, Rh, and bacterial cell extracts from the R, Stem, and Leaf samples were used for DNA extraction. Total DNA was extracted using the DNeasy Power Soil kit (QIAGEN) according to the manufacturer’s protocol.

### 16S amplicon sequence ana­lysis of the bacterial community of water yam

We targeted the hypervariable V3–V4 regions of bacterial 16S rRNA genes to analyze the bacterial community of water yam. The V3–V4 regions of bacterial 16S rRNA were amplified using the following primer pairs: forward, 5′-TCGTCGGCAGCGTCAGATGTGTATAAGAGACAGCCTACGGGNGGCWGCAG-3′ and reverse, 5′-GTCTCGTGGGCTCGGAGATGTGTATAAGAGACAGGACTACHVGGGTATC TAATCC-3′. The nucleotide sequences of the Illumina adapter overhang are shown as underlined regions, while non-underlined sequences are gene-specific sequences targeting the V3–V4 regions of the prokaryotic 16S rRNA genes (Klindworth *et al.*, 2013). A PCR amplicon library was constructed following the Illumina16S sample preparation guide (16S Sample Preparation Guide, 15044223; Illumina). The 300-bp paired-end sequencing of libraries was performed using a MiSeq sequencer (Illumina). All sequenced data obtained in the present study were deposited in the DDBJ Sequence Read Archive (DRA) database (https://www.ddbj.nig.ac.jp/dra/index-e.html) under accession number DRA012357.

The sequence reads of all samples were processed using QIIME2 (Bolyen *et al.*, 2019). Due to quality control, only forward amplicon reads were used in the present study. FASTQ files were quality filtered, trimmed, and de-noised, and chimeric sequences were removed and merged using DADA2 (Callahan *et al.*, 2016) in QIIME2 (version 2018.11). Sequences were clustered into amplicon sequence variants (ASVs) with 100% identity. The taxonomic classification of ASVs was performed using the SILVA132 database (Quast *et al.*, 2013). ASVs classified under chloroplasts or mitochondria were removed for further ana­lyses.

### Statistical ana­lysis

The dry weights of plants were compared using Welch’s *t*-test. Statistical ana­lyses and the visualization of ASV data were performed using QIIME2 (Bolyen *et al.*, 2019), Calypso software (Zakrzewski *et al.*, 2017), and R software version 4.1.1 (R Core Team, 2021) using the packages iNEXT (Hsieh *et al.*, 2016), tidyverse (Wickham *et al.*, 2019), dunn.test (Dinno, 2017), and vegan (Oksanen *et al.*, 2020). Sample size-based rarefaction and coverage-based rarefaction (Chao and Jost, 2012) were performed for alpha diversity measures based on the ASV table with the iNEXT package (Hsieh *et al.*, 2016) using 50 bootstrap replicates per sample. The sample size-based rarefaction curve did not plateau at the same sample size for all samples (Fig. S1a and b). Therefore, species richness and Shannon-based effective number of species (ENS) indices (Chao *et al.*, 2014) were calculated using coverage-based rarefaction at a coverage of 98.5%, which corresponded to the lowest coverage calculated for a sample using the iNEXT package (Hsieh *et al.*, 2016). The effects of the fertilizer treatment on each of the alpha diversity indices for each plant compartment were assessed using the Kruskal-Wallis test, followed by Dunn’s test with Bonferroni corrections in R (Dinno, 2017). The relationship between the bacterial community structure and each plant part was visualized using a principal coordinate ana­lysis (PCoA) based on the weighted UniFrac distance matrix calculated from the raw ASV abundance table (non-rarefied) using QIIME2. The effects of fertilization and plant compartments on the bacterial community were assessed using a permutational ana­lysis of variance (PERMANOVA; Adonis function; 999 permutations) with the vegan package (Oksanen *et al.*, 2020). Heatmap and community composition ana­lyses were performed using Calypso with no additional filtering and total sum normalization.

## Results

### Effects of fertilization on the growth of water yam

The dry weights of the leaves, stems, and roots of treated and control water yam cv. A-19 are shown in Table 1. The application of urea did not significantly affect the dry weight of water yam cv. A-19 (*t*-test, *P*<0.05).

### Characteristics of amplicon sequence data on water yam

To obtain a broad picture of the microbial community of water yam, we analyzed 30 samples (five plant compartments [BS, Rh, R, Stem, and Leaf]×two fertilization regimes [with and without chemical fertilization]×three replicates). We generated 5,362,844 paired-end reads using the Illumina MiSeq sequencing platform (average: 178,761; range: 10,119–393,304 reads per sample; Table S1). After quality filtering, denoising, the merging of pair-end reads, and chimera screening, 4,253,396 reads were retained. Following the removal of ASVs representing singletons and those classified as chloroplasts or mitochondria, 3,011,241 reads were retained, comprising 70.8% of the original sequences (average: 100,375; range: 292–300,049 reads per sample) and 6,049 ASVs.

### Alpha diversity of the bacterial community of plant compartments

**Fig. 1.**
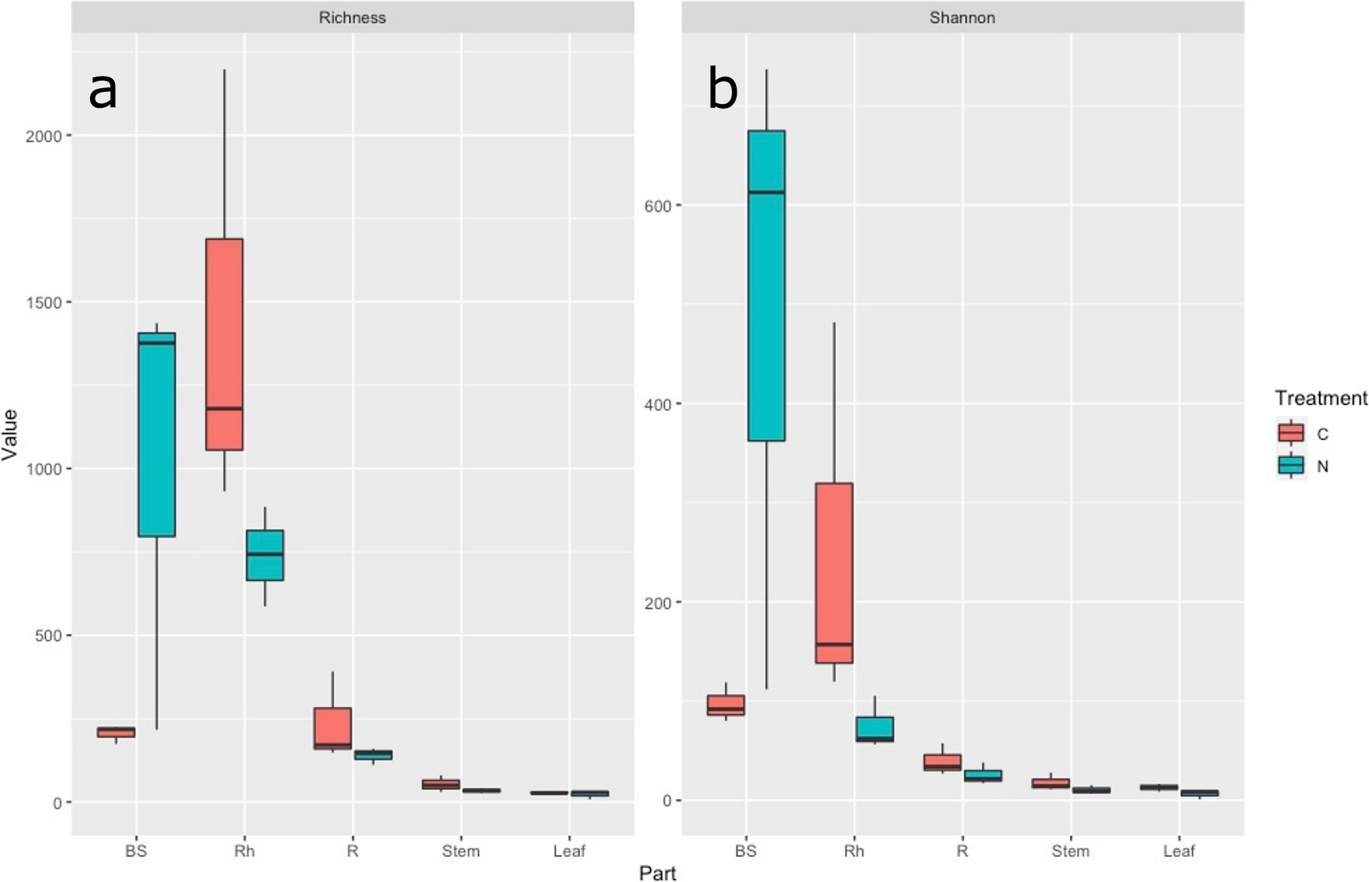
Alpha diversity indices for 16S rRNA gene sequences. Box plots of species richness (a), and Shannon-based ENS indices (b) in Bulk soil (BS), Rhizosphere (Rh), Root (R), Stem, and Leaf in the control (C) and nitrogen treatment (N). The Kruskal-Wallis test followed by Dunn’s test with Bonferroni corrections was used to assess the effects of chemical fertilization on indices.

Microbial diversity across plant compartments and fertilization regimes was evaluated by analyzing alpha diversity using species richness and Shannon-based ENS indices based on coverage-based rarefaction to a coverage of 98.5% (Fig. 1a and b). The species richness index was highly dependent on the plant compartment (the Kruskal-Wallis test followed by Dunn’s test *P*<0.01), with high richness values for Rh samples (average values of 1,436.11 for the control and 738.25 for the treatment) and consistently low richness values for BS (205.26 for the control and 1,009.48 for the treatment), R (236.66 for the control and 138.96 for the treatment), Stem (53.38 for the control and 33.82 for the treatment), and Leaf samples (27.97 for the control and 22.74 for the treatment). Similar results were observed for the Shannon-based ENS index. The fertilizer treatment did not significantly affect bacterial diversity indices (the Kruskal-Wallis test followed by Dunn’s test *P*>0.05).

### Beta diversity for the bacterial community structure of plant compartments

**Fig. 2.**
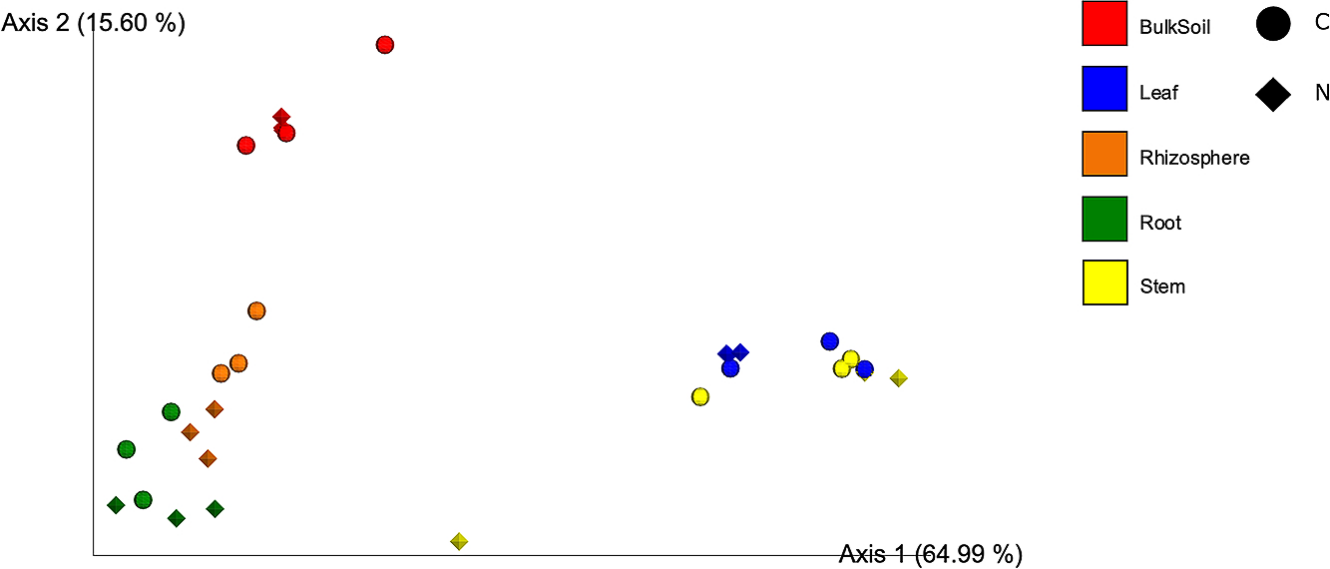
Principal coordinate ana­lysis of bacterial communities in different plant compartments based on the weighted UniFrac distance on Bulk soil, Rhizosphere, Root, Stem, and Leaf. C, control; N, nitrogen treatment

To assess whether microbiomes formed distinct communities when grouped by plant compartments and fertilizer treatments, we used PCoA to visualize differences between bacterial communities (beta diversity) based on the weighted UniFrac distance computed on the non-rarefied dataset at the ASV level (Fig. 2). PCoA revealed that bacterial communities were separated by plant compartments (PERMANOVA, R^2^=0.852, *P*<0.01), but not by treatment (PERMANOVA, R^2^=0.010, *P*=0.174). The fertilizer treatment did not significantly affect the overall community composition.

### Bacterial community of plant compartments with different fertilization practices

**Fig. 3.**
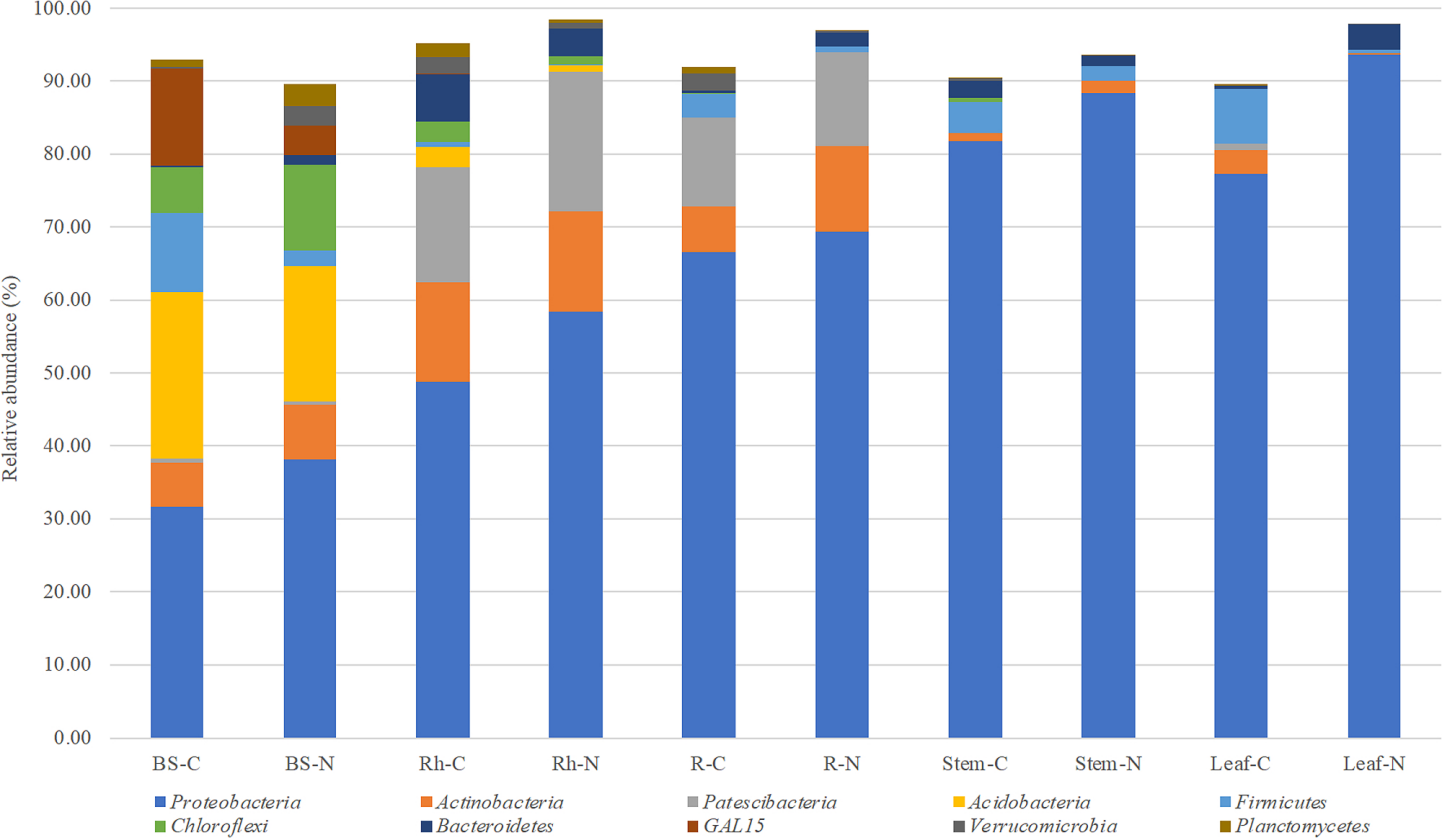
Average relative abundance of top 10 phyla in different plant compartments. BS-C, Bulk soil on control; BS-N, Bulk soil on the nitrogen treatment; Rh-C, Rhizosphere on the control; Rh-N, Rhizosphere on the nitrogen treatment; R-C, Root on the control; R-N, Root on the nitrogen treatment; Stem-C, Stem on the control; Stem-N, Stem on the nitrogen treatment; Leaf-C, Leaf on the control; Leaf-N, Leaf on the nitrogen treatment.

We investigated the bacterial members present in communities associated with the different plant compartments of water yam. We detected 39 phyla in each plant compartment. The average relative abundance of the top 10 phyla (0.78 to 65.41%) is shown in Fig. 3 and Table S2.

**Fig. 4.**
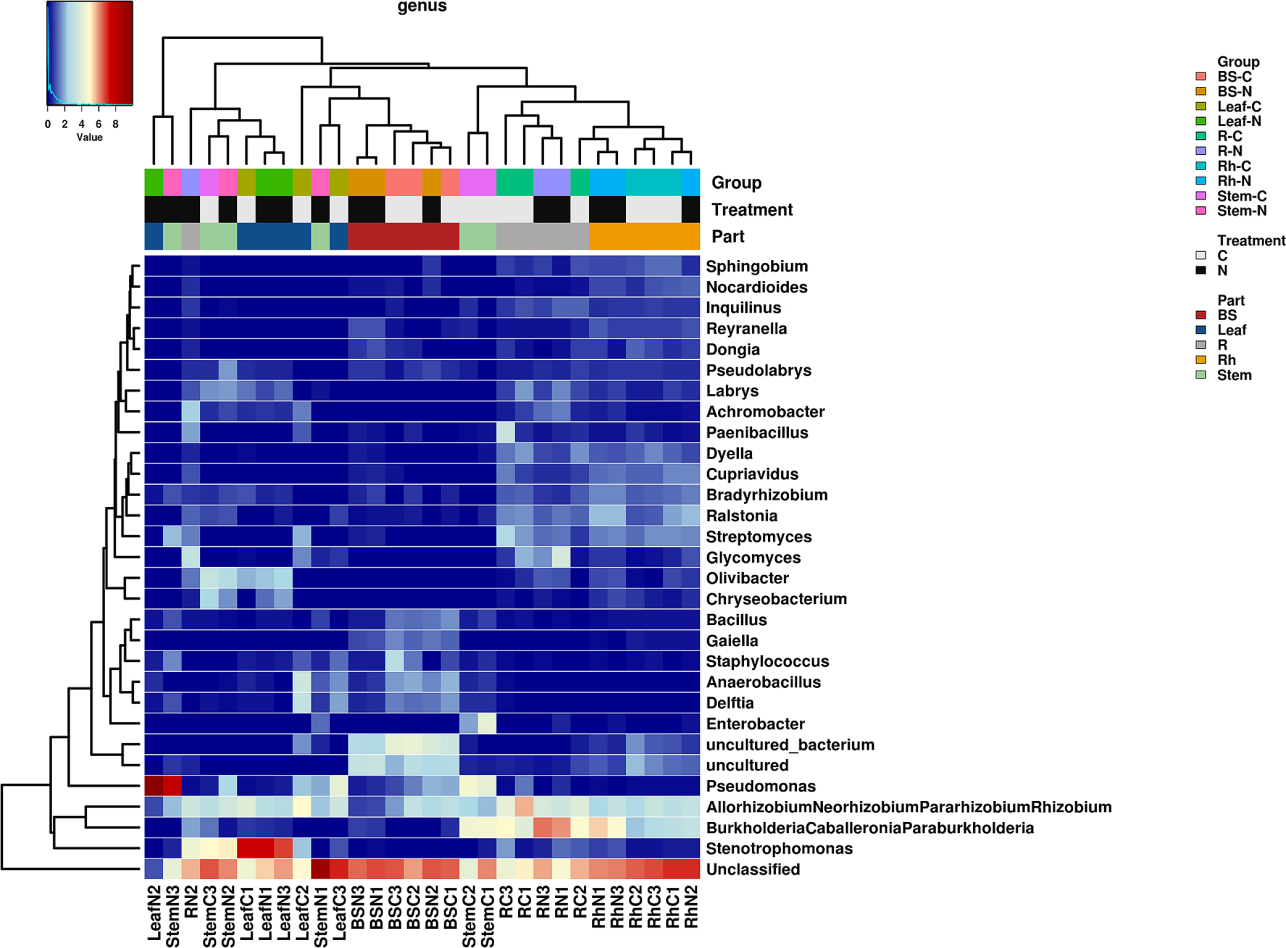
Heatmap of bacterial distribution of top 30 abundant genera on all compartments. The heatmap color (blue to red) represents the relative abundant genera from low to high. BS-C, Bulk soil on the control; BS-N, Bulk soil on the nitrogen treatment; Rh-C, Rhizosphere on the control; Rh-N, Rhizosphere on the nitrogen treatment; R-C, Root on the control; R-N, Root on the nitrogen treatment; Stem-C, Stem on the control; Stem-N, Stem on the nitrogen treatment; Leaf-C, Leaf on the control; Leaf-N, Leaf on the nitrogen treatment.

Based on the average relative abundance in each of the plant compartments, *Proteobacteria* was the predominant phylum (31.70 to 93.70%), followed by *Actinobacteria* and *Patescibacteria* (>5% average relative abundance). The subdominant phyla (>1% average relative abundance) were *Acidobacteria*, *Firmicutes*, *Chloroflexi*, *Bacteroidetes*, and *GAL15*. In addition, we detected 533 genera in each plant compartment (Table S3). To identify microbial distribution patterns across plant compartments, the top 30 abundant genera (>0.22% average relative abundance) are shown in Fig. 4 and Table S4. We described the dominant genera (>*ca.* 1% average relative abundance in at least one compartment), excluding unclassified and uncultured genera. The top seven most abundant genera, the *Allorhizobium-Neorhizobium-Pararhizobium-Rhizobium* clade, *Burkholderia-Caballeronia-Paraburkholderia* clade, *Stenotrophomonas*, *Pseudomonas*, *Glycomyces*, *Ralstonia*, and *Streptomyces*, were predominant in all compartments (>1% average relative abundance).

The dominant genera in BS were the *Allorhizobium*-*Neorhizobium*-*Pararhizobium*-*Rhizobium* clade, *Anaerobacillus*, *Pseudomonas*, *Staphylococcus*, *Gaiella*, *Delftia*, and *Bacillus*. The relative abundance of *Staphylococcus* significantly differed between the treatment and control (ANOVA followed by Tukey’s HSD post hoc test, *P*<0.05). The dominant genera in Rh were the *Burkholderia*-*Caballeronia*-*Paraburkholderia* clade, *Allorhizobium*-*Neorhizobium*-*Pararhizobium*-*Rhizobium* clade, *Ralstonia*, *Streptomyces*, *Bradyrhizobium*, *Cupriavidus*, *Dyella*, and *Kribbella*. Although the abundance of these genera did not significantly differ between the treatment and control, the *Burkholderia*-*Caballeronia*-*Paraburkholderia* clade was slightly more abundant in the fertilizer treatment.

In R, the dominant genera were the *Burkholderia*-*Caballeronia*-*Paraburkholderia* clade, *Allorhizobium*-*Neorhizobium*-*Pararhizobium*-*Rhizobium* clade, *Glycomyces*, *Stenotrophomonas*, *Streptomyces*, *Ralstonia*, *Achromobacter*, *Dyella*, *Paenibacillus*, *Labrys*, *Bradyrhizobium*, *Inquilinus*, *Cupriavidus*, and *Olivibacter*. The relative abundance of *Glycomyces*, *Achromobacter*, and *Dyella* significantly differed between the fertilizer treatment and control (*P*<0.05).

The dominant genera in Stem were the *Pseudomonas*, *Allorhizobium*-*Neorhizobium*-*Pararhizobium*-*Rhizobium* clade, *Burkholderia*-*Caballeronia*-*Paraburkholderia* clade, *Stenotrophomonas*, *Enterobacter*, *Olivibacter*, and *Lactococcus*. The relative abundance of the *Burkholderia*-*Caballeronia*-*Paraburkholderia* clade significantly differed between the fertilizer treatment and control (*P*<0.05).

The dominant genera in Leaf were *Stenotrophomonas*, the *Allorhizobium-Neorhizobium-Pararhizobium-Rhizobium* clade, *Pseudomonas*, *Anaerobacillus*, *Delftia*, *Olivibacter*, and *Chryseobacterium*. The relative abundance of *Delftia* significantly differed between the fertilizer treatment and control (*P*<0.05).

Among the dominant genera, the relative abundance of the following five genera significantly differed between the fertilizer treatment and control, excluding bulk soil. Specifically, the relative abundance of *Dyella* in R, *Delftia* in Leaf, and the *Burkholderia-Caballeronia-Paraburkholderia* clade in Stem were higher in the control than in the fertilizer treatment. In contrast, the relative abundance of *Glycomyces* and *Achromobacter* in R was lower in the control than in the fertilizer treatment. Furthermore, due to high variability in abundance between replicates, the relative abundance of various genera only slightly differed between the fertilizer treatment and control.

## Discussion

To obtain a more detailed understanding of crop growth and the endophytic community of water yam, we herein examined the bacterial community of water yam cultivated with and without chemical fertilization, and the influence of fertilization practices on the composition of the bacterial community. To the best of our knowledge, this is the first study to investigate the community profile of water yam and the influence of fertilization practices on the composition of the bacterial community. No significant differences were observed in the growth of water yam cultivated with or without chemical fertilization (Table 1), which is consistent with previous findings (Shiwachi *et al.*, 2015; Rezaei *et al.*, 2016, 2017; Takada *et al.*, 2017, 2018; Ouyabe *et al.*, 2019a, 2019b). 

In plants affected by PGPB, Ikeda *et al.* (2014) reported that the growth of rice plants markedly decreased when cultivated with low nitrogen fertilization. They also showed that while low nitrogen fertilization markedly reduced the growth of rice, specific bacteria within the endophytic community appeared to contribute to the plant’s ability to adapt to its environment, thereby facilitating continued growth. These findings prompted us to hypothesize that the abundance of specific bacteria in the endophytic community of water yam cv. A-19 changes depending on fertilization in order to promote continued plant growth under low fertile conditions.

The amplicon profiling of whole plant compartments of water yam cultivated with and without chemical fertilization was performed in the present study. Alpha diversity indices were significantly dependent on each of the compartments; a decrease was noted in the indices from the belowground to aboveground compartments (Fig. 1a and b). Chemical fertilization did not significantly influence the alpha diversity of the bacterial community. PCoA of the beta diversity plot revealed three compartment-based clusters: BS, Rh, and R, while Stem and Leaf (Fig. 2) were not clustered by fertilization. These results on the composition of bacterial community in water yam were consistent with previous findings on plant fine-tuning assemblages, called the two-step model (Bulgarelli *et al.*, 2013). This model proposes that the community composition is strongly influenced by soil conditions as the primary bacterial source. The compositions of the bacterial communities in the root, rhizosphere, and phyllosphere compartments significantly differ from each other and from that in the soil, with a rhizoplane that serves as a gating point for controlling bacterial entry into the endosphere.

Overall, the bacterial diversity and community structure remained robust in the presence of chemical fertilization; however, the relative abundance of some genera changed in response to the fertilizer treatment. Among the dominant genera, the relative abundance of five genera significantly differed between the fertilizer treatment and control, with only one genus (the *Burkholderia*-*Caballeronia*-*Paraburkholderia* clade in Stem) being more abundant in the control (Table S4). Therefore, bacteria belonging to this clade may affect the growth of water yam under low fertile conditions.

Among the top 30 bacterial genera of water yam, seven were predominant (Fig. 4 and Table S4). Among them, the *Allorhizobium*-*Neorhizobium*-*Pararhizobium*-*Rhizobium* clade, *Burkholderia*-*Caballeronia*-*Paraburkholderia* clade, *Stenotrophomonas*, *Pseudomonas*, *Ralstonia*, and *Streptomyces*, belonging to *Proteobacteria*, *Actinobacteria*, and *Firmicutes*, are PGPB (Perin *et al.*, 2006; Mano *et al.*, 2007; Terakado-Tonooka *et al.*, 2008, 2013; Taulé *et al.*, 2012; Ikeda *et al.*, 2014; Madhaiyan *et al.*, 2015; Sathya *et al.*, 2017) (Table S5). In addition, these genera were isolated as PGPB from yam (Rezaei *et al.*, 2017; Ouyabe *et al.*, 2019a, 2019b, 2019c; Takada *et al.*, 2019; Shiwachi *et al.*, 2020) and are regarded as the most common genera among the bacterial community of water yam. Among the six genera, two bacterial clades, the *Allorhizobium*-*Neorhizobium*-*Pararhizobium*-*Rhizobium* clade and *Burkholderia*-*Caballeronia*-*Paraburkholderia* clade, were predominant, particularly in R (>20% of abundance) cultivated with and without chemical fertilization (Table S4). This is a unique feature of water yam over other crops, such as sugarcane and rice (Ikeda *et al.*, 2014; Yeoh *et al.*, 2016). The relative abundance of *Burkholderia* sp. varies with nitrogen levels in rice; it was higher (>20%) at low nitrogen levels than at standard or high nitrogen fertilization levels in the root endophytic community (*ca.* 1%) (Ikeda *et al.*, 2014).

The common bacterial genera of tuber crops may be inherited from previous generations, similar to the soil bacterial community (Buchholz *et al.*, 2019). Alternatively, vertical transmission of the bacterial community of water yam from tubers has also been reported (Shiwachi *et al.*, 2020). In the endophytic community of water yam Stem and Leaf samples in the present study, the abundance of *Stenotrophomonas* and *Pseudomonas* increased, similar to previous findings on endophytic communities within stem and leaf samples of lettuce, poplar, sugarcane, and potato (Becker *et al.*, 2008; Taulé *et al.*, 2012; Jackson *et al.*, 2013; Beckers *et al.*, 2017). Moreover, the abundance of the *Allorhizobium*-*Neorhizobium*-*Pararhizobium*-*Rhizobium* clade was high, while that of the *Burkholderia*-*Caballeronia*-*Paraburkholderia* clade was low in the endophytic community of Stem and Leaf in water yam. A previous study that performed amplicon profiling of the phyllospheric community in aerial yam (*D. bulbifera* L.) reported that a new isolate, *Paraburkholderia* sp., with plant growth-promoting traits accounted for >25% of the endophytic community abundance in the leaf acumen (Herpell *et al.*, 2020). The present results suggest that the bacterial communities represented by these two clades were inherited through tubers from previous generations to promote the growth of water yam under low fertile conditions.

In the present study, the *Burkholderia*-*Caballeronia*-*Paraburkholderia* clade was not enriched in the endophytic community of water yam Leaf. In contrast, Ouyabe *et al.* (2019a) reported that 76% of isolated bacteria from lesser yam (*D. esculenta* L.), including *Burkholderia* sp. and *Paraburkholderia* sp., were associated with the phyllosphere of the plant. This result suggests that the bacterial community varies among yam varieties.

A previous culture-dependent ana­lysis showed variations in the composition of the bacterial community among yam varieties (Ouyabe *et al.*, 2019a), and demonstrated that the bacterial community composition of the lesser yam dominated the phyllosphere, in contrast to that of water yam in the present study, which dominated the endosphere of the root. Moreover, the bacterial community structure of water yam did not significantly differ between the chemical fertilization treatment and the control. The abundance of four genera of water yam, including the *Burkholderia*-*Caballeronia*-*Paraburkholderia* clade in the Stem, cultivated with and without chemical fertilization significantly differed. Caradonia *et al.* (2019) reported that different applications of nitrogen fertilizers (chemical or organic) induced complementary patterns in the bacterial community of tomatoes, producing a “distinct signature” represented by *Actinobacteria* in root communities. However, similar to the present results, they found no significant changes in the alpha diversity index between nitrogen fertilization levels. The present results suggest that the effects of urea application were more subtle than other experimental factors, such as soil conditions, microbial assemblage by the plant, and the sampling season (Yeoh *et al.*, 2016). Therefore, the difference in the *Burkholderia*-*Caballeronia*-*Paraburkholderia* clade in Stem and Leaf was attributed to variations among yam varieties. In this experiment, we observed the effects of the chemical fertilizer on the bacterial community of one yam variety. However, further studies are needed to elucidate the effects of organic fertilization and soil fertility on the function of the bacterial community in yam, which may affect plant growth.

## Citation

Kihara, S., Yamamoto, K., Hisatomi, A., Shiwa, Y., Chu, C.-C., Takada, K., et al. (2022) Bacterial Community of Water Yam (*Dioscorea alata* L.) cv. A-19. *Microbes Environ ***37**: ME21062.

https://doi.org/10.1264/jsme2.ME21062

## Supplementary Material

Supplementary Material

## Figures and Tables

**Table 1.** Effects of nitrogen application on the growth of water yam cv. A-19 120 days after planting

Treatment	Dry weight of leaves (g)	Dry weight of stems (g)	Dry weight of roots (g)
Control	24.32±8.93^n.s.^	18.50±11.84^n.s.^	12.47±11.27^n.s.^
Treatment	30.65±19.97	25.06±16.37	7.13±7.78

The experiment was conducted using urea application as nitrogen at a rate equivalent to 30‍ ‍kg 10 a^–1^ as treatment at 60 days after planting (DAP), and without nitrogen application as a control. Samples were collected 120 DAP. n.s. indicates no significant difference according to Welch’s *t*-test (*P*<0.05). Values represent the mean of five replicates±SD.
