# Supplementary material for: Bacterial Community of Water Yam (Dioscorea alata L.) cv. A-19
Source: Microbes Environ. 2022 May 3;37(2):ME21062. doi: 10.1264/jsme2.ME21062 (PMC9530735; doi:10.1264/jsme2.ME21062)
Supplement: Supplementary file 1 — Supplementary Material [file 37_21062_s1.pdf]

Supplemental material

Bacterial community of water yam (*Dioscorea alata* L.) cv. A-19

Shunta Kihara, Kosuke Yamamoto, Atsushi Hisatomi, Yuh Shiwa*, Chia-Cheng Chu, Kanako Takada, Michel Ouyabe, Babil Pachakkil, Hidehiko Kikuno, Naoto Tanaka, and Hironobu Shiwachi

Table S1. Characteristics of the effective tags from samples of bacteria from each compartment associated with water yam (*Dioscorea alata* L.) cv.A-19 cultivated with levels of ferilizers.

Sample ID	Description<br>C: control<br>N: nitrogen treatment	# input reads	# reads after quality filtering	# reads after quality denoising	# non-chimeric reads	# reads after filtering of chloroplast and mitochondria reads	# ASV
YM8-1-1	BS-C-1	15,162	11,708	11,708	11,667	11,655	233
YM8-1-2	BS-C-2	11,438	8,904	8,904	8,898	8,851	187
YM8-1-3	BS-C-3	24,286	18,514	18,514	18,459	18,442	247
YM8-1-4	Rh-C-1	393,304	320,807	320,807	310,581	295,886	1,522
YM8-1-5	Rh-C-2	329,677	266,600	266,600	260,364	244,791	2,554
YM8-1-6	Rh-C-3	142,107	116,730	116,730	114,608	107,989	1,097
YM8-1-7	R-C-1	187,395	157,354	157,354	155,842	151,264	258
YM8-1-8	R-C-2	313,713	266,552	266,552	258,503	257,603	617
YM8-2-1	R-C-3	117,086	96,224	96,224	94,993	91,193	270
YM8-2-2	Leaf-C-1	150,581	123,959	123,959	123,112	19,829	53
YM8-2-3	Leaf-C-2	121,566	97,959	97,959	97,857	292	27
YM8-2-4	Leaf-C-3	159,321	127,118	127,118	126,682	549	27
YM8-2-5	Stem-C-1	145,482	116,543	116,543	116,492	9,211	68
YM8-2-6	Stem-C-2	142,116	112,446	112,446	112,081	8,488	98
YM8-2-7	Stem-C-3	129,956	102,652	102,652	101,683	25,211	53
YM8-2-8	BS-N-1	107,546	83,011	83,011	80,179	80,095	1,546
YM8-3-1	BS-N-2	10,119	7,816	7,816	7,795	7,724	231
YM8-3-2	BS-N-3	130,124	101,835	101,835	97,944	97,920	1,505
YM8-3-3	Rh-N-1	286,575	235,963	235,963	229,561	219,606	1,009
YM8-3-4	Rh-N-2	277,517	226,789	226,789	222,344	209,856	1,138
YM8-3-5	Rh-N-3	361,736	296,148	296,148	289,244	269,792	874
YM8-3-6	R-N-1	303,839	250,876	250,876	243,120	239,378	287
YM8-3-7	R-N-2	325,890	239,619	239,619	234,247	221,478	283
YM8-3-8	R-N-3	377,382	316,821	316,821	302,075	300,049	254
YM8-4-1	Leaf-N-1	174,933	139,967	139,967	138,767	21,171	53
YM8-4-2	Leaf-N-2	90,487	72,784	72,784	72,784	14,984	31
YM8-4-3	Leaf-N-3	153,914	122,263	122,263	121,392	16,268	49
YM8-4-4	Stem-N-1	106,885	83,999	83,999	83,939	3,878	50
YM8-4-5	Stem-N-2	129,191	105,381	105,381	103,685	56,709	56
YM8-4-6	Stem-N-3	143,516	114,800	114,800	114,498	1,079	37
	Total	5,362,844	4,342,142	4,342,142	4,253,396	3,011,241	14,714
	Min	10,119	7,816	7,816	7,795	292	27
	Max	393,304	320,807	320,807	310,581	300,049	2,554
	Average	178,761	144,738	144,738	141,780	100,375	490

Characteristics of the effective tags from samples of bacteria from each compartment associated with water yam (Dioscorea alata L.) cv. A-19 cultivated with levels of fertilizers. BS-C, Bulk soil on control; BS-N, Bulk soil on nitrogen treatment; Rh-C, Rhizosphere on control; Rh-N, Rhizosphere on nitrogen treatment; R-C, Root on control; R-N, Root on nitrogen treatment; Stem-C, Stem on control; Stem-N, Stem on nitrogen treatment; Leaf-C, Leaf on control; Leaf-N, Leaf on nitrogen treatment.

Table S2. Relative abundances (% of total good-quality sequences) of top 10 phyla (0.78 to 65.41%) in each compertment of water yam (*Dioscorea alata* L.) cv.A-19 cultivated with levels of fertilizers.

Phylum	Average	BS-C	BS-N	Rh-C	Rh-N	R-C	R-N	Stem-C	Stem-N	Leaf-C	Leaf-N
Proteobacteria	65.41	31.70	38.19	48.86	58.37	66.59	69.39	81.72	88.36	77.26	93.70
Actinobacteria	6.50	6.00	7.51	13.62	13.76	6.30	11.63	1.10	1.64	3.35	0.13
Patescibacteria	6.16	0.53	0.38	15.76	19.10	12.04	12.89	0.03	0.03	0.83	0.00
Acidobacteria	4.52	22.85	18.58	2.69	0.96	0.08	0.03	0.04	0.00	0.00	0.00
Firmicutes	3.22	10.89	2.16	0.69	0.18	3.24	0.85	4.24	2.07	7.45	0.40
Chloroflexi	2.24	6.25	11.70	2.86	1.00	0.11	0.03	0.46	0.00	0.00	0.00
Bacteroidetes	2.15	0.10	1.25	6.50	3.84	0.28	1.74	2.43	1.49	0.44	3.46
GAL15	1.75	13.32	4.08	0.08	0.01	0.00	0.00	0.00	0.00	0.00	0.00
Verrucomicrobia	0.90	0.27	2.72	2.22	0.71	2.40	0.26	0.37	0.00	0.00	0.00
Planctomycetes	0.78	1.06	3.06	1.85	0.50	0.86	0.14	0.01	0.05	0.27	0.00

>20%

>10%

>5%

>1%

Relative abundances (% of total good-quality sequences) of top 10 phyla (0.78 to 65.41%) in each compartment of water yam (*Dioscorea alata* L.) cv. A-19 cultivated with different levels of fertilizers. BS-C, Bulk soil on control; BS-N, Bulk soil on nitrogen treatment; Rh-C, Rhizosphere on control; Rh-N, Rhizosphere on nitrogen treatment; R-C, Root on control; R-N, Root on nitrogen treatment; Stem-C, Stem on control; Stem-N, Stem on nitrogen treatment; Leaf-C, Leaf on control; Leaf-N, Leaf on nitrogen treatment.

Table S3.Relative abundance (%) of bacterial genera found through 16S rRNA gene amplicon profiling of DNA extracted from each compartment of water yam (*Dioscorea alata* L.) cv.A-19 cultivated with levels of ferilizers. BS-C, Bulk soil on control; BS-N, Bulk soil on nitrogen treatment; Rh-C, Rhizosphere on control; Rh-N, Rhizosphere on nitrogen treatment; R-C, Root on control; R-N, Root on nitrogen treatment; Stem-C, Stem on control; Stem-N, Stem on nitrogen treatment; Leaf-C, Leaf on control; Leaf-N, Leaf on nitrogen treatment.

	One-way ANOVA				P values of Tukey's HSD post hoc					Average relative abundance among plant compartments and fertilizer treatments (C: control, N: fertilized)										
Taxa	P Group	FDR Group	P adjusted Group	F Value Group	BS	Rh	R	Stem	Leaf	Average	BS-C mean	BS-N mean	Rh-C mean	Rh-N mean	R-C mean	R-N mean	Stem-C mean	Stem-N mean	Leaf-C mean	Leaf-N mean
Unclassified	0.390	0.560	1.000	1.100	1.000	1.000	1.000	1.000	0.950	34.140	39.564	42.510	44.756	40.832	25.301	29.160	31.472	41.861	29.703	16.241
AllorhizobiumNeorhizobium PararhizobiumRhizobium	0.002	0.008	0.730	4.800	0.620	1.000	0.720	1.000	0.090	9.404	6.760	1.960	8.880	8.352	21.160	12.250	6.300	6.864	16.892	4.623
BurkholderiaCaballeroniaParaburkholderia	0.000	0.000	0.004	10.000	1.000	0.590	1.000	0.029	1.000	8.153	0.036	0.230	6.760	19.448	20.976	21.902	12.041	0.005	0.044	0.090
Stenotrophomonas	0.230	0.410	1.000	1.500	1.000	1.000	1.000	1.000	1.000	4.669	0.000	0.004	0.090	0.436	0.980	4.203	3.028	3.423	12.532	21.996
Pseudomonas	0.320	0.560	1.000	1.300	1.000	1.000	1.000	1.000	1.000	4.565	2.016	0.941	0.017	0.014	0.260	0.058	9.120	16.403	4.709	12.110
uncultured_bacterium	0.000	0.000	0.000	30.000	0.260	0.820	1.000	1.000	0.850	2.695	15.682	9.120	1.416	0.360	0.029	0.003	0.012	0.026	0.303	0.000
uncultured	0.000	0.000	0.000	40.000	0.730	0.200	1.000	1.000	1.000	1.945	6.503	8.880	2.624	0.903	0.292	0.123	0.073	0.053	0.000	0.000
Glycomyces	0.000	0.000	0.011	9.100	1.000	0.980	0.006	1.000	0.670	1.008	0.000	0.000	0.096	0.578	0.903	7.896	0.000	0.020	0.593	0.000
Ralstonia	0.000	0.000	0.000	15.000	1.000	0.079	0.990	1.000	1.000	1.007	0.084	0.012	1.588	4.494	2.103	1.440	0.176	0.102	0.063	0.010
Streptomyces	0.009	0.027	1.000	3.600	1.000	1.000	1.000	0.920	0.940	1.007	0.000	0.073	2.220	2.280	2.856	1.690	0.000	0.490	0.462	0.000
Anaerobacillus	0.001	0.006	0.520	5.100	0.450	1.000	1.000	1.000	0.062	0.794	3.610	0.757	0.001	0.001	0.004	0.000	0.123	0.152	3.240	0.058
Olivibacter	0.580	0.600	1.000	0.850	1.000	1.000	0.980	1.000	0.970	0.699	0.000	0.000	0.130	0.624	0.063	1.254	1.188	0.828	0.436	2.465
Delftia	0.002	0.010	0.890	4.600	0.800	1.000	1.000	1.000	0.027	0.622	2.103	0.578	0.001	0.000	0.005	0.000	0.168	0.314	3.028	0.026
Bradyrhizobium	0.001	0.003	0.260	5.700	1.000	0.880	0.770	0.860	1.000	0.605	0.137	0.168	1.254	2.280	1.166	0.410	0.036	0.348	0.102	0.152
Dyella	0.000	0.000	0.000	47.000	0.810	0.250	0.000	1.000	1.000	0.557	0.000	0.040	1.664	0.922	2.434	0.504	0.009	0.000	0.002	0.000
Cupriavidus	0.000	0.000	0.000	25.000	0.980	1.000	0.550	1.000	1.000	0.503	0.006	0.063	1.613	1.904	1.000	0.449	0.000	0.000	0.000	0.000
Labrys	0.320	0.560	1.000	1.200	1.000	1.000	1.000	1.000	1.000	0.440	0.000	0.000	0.221	0.360	1.210	1.166	0.303	0.423	0.212	0.504
Enterobacter	0.054	0.120	1.000	2.300	1.000	1.000	1.000	0.220	1.000	0.419	0.000	0.000	0.001	0.017	0.000	0.036	3.960	0.176	0.000	0.000
Staphylococcus	0.008	0.027	1.000	3.600	0.030	1.000	1.000	0.980	0.900	0.417	2.723	0.053	0.029	0.009	0.004	0.002	0.084	0.476	0.706	0.090
Achromobacter	0.001	0.007	0.560	5.000	1.000	1.000	0.038	1.000	0.990	0.388	0.000	0.000	0.020	0.137	0.281	2.723	0.040	0.078	0.449	0.152
Paenibacillus	0.180	0.330	1.000	1.600	1.000	1.000	0.970	1.000	1.000	0.333	0.023	0.036	0.123	0.068	2.161	0.774	0.026	0.000	0.116	0.000
Bacillus	0.000	0.000	0.022	8.300	0.052	1.000	1.000	1.000	0.980	0.330	2.016	0.490	0.073	0.044	0.036	0.020	0.203	0.372	0.000	0.048
Gaiella	0.000	0.000	0.000	62.000	0.250	0.600	1.000	1.000	1.000	0.281	1.613	1.082	0.096	0.017	0.000	0.000	0.000	0.000	0.000	0.000
Sphingobium	0.000	0.000	0.000	17.000	0.900	0.180	0.380	1.000	1.000	0.268	0.000	0.044	1.346	0.548	0.578	0.168	0.000	0.000	0.000	0.000
Chryseobacterium	0.440	0.560	1.000	1.000	1.000	1.000	1.000	1.000	0.600	0.257	0.000	0.000	0.168	0.397	0.014	0.073	0.624	0.292	0.000	1.000
Inquilinus	0.000	0.000	0.000	18.000	1.000	1.000	1.000	0.990	1.000	0.247	0.010	0.000	0.476	0.384	0.884	0.689	0.029	0.002	0.000	0.000
Pseudolabrys	0.750	0.750	1.000	0.640	1.000	1.000	1.000	0.990	1.000	0.247	0.221	0.490	0.348	0.384	0.230	0.260	0.084	0.336	0.029	0.084
Kribbella	0.000	0.000	0.000	26.000	0.084	0.075	1.000	1.000	1.000	0.222	0.000	0.137	0.640	1.392	0.012	0.040	0.000	0.000	0.000	0.000
Niastella	0.000	0.000	0.000	54.000	0.026	0.001	1.000	1.000	1.000	0.216	0.014	0.185	1.416	0.548	0.000	0.000	0.000	0.000	0.000	0.000

	One-way ANOVA				P values of Tukey’s HSD post hoc					Average relative abundance among plant compartments and fertilizer treatments (C: control, N: fertilized)										
Taxa	P Group	FDR Group	P adjusted Group	F Value Group	BS	Rh	R	Stem	Leaf	Average	BS-C mean	BS-N mean	Rh-C mean	Rh-N mean	R-C mean	R-N mean	Stem-C mean	Stem-N mean	Leaf-C mean	Leaf-N mean
Lactococcus	0.540	0.570	1.000	0.900	1.000	1.000	1.000	0.710	1.000	0.216	0.325	0.002	0.000	0.000	0.000	0.000	1.716	0.032	0.078	0.004
Reyranella	0.000	0.001	0.054	7.200	0.340	1.000	1.000	1.000	1.000	0.201	0.048	0.423	0.563	0.792	0.109	0.058	0.023	0.000	0.000	0.000
Nocardioides	0.000	0.000	0.000	19.000	0.640	1.000	1.000	1.000	1.000	0.181	0.012	0.109	0.723	0.846	0.040	0.078	0.000	0.000	0.000	0.000
Dongia	0.002	0.010	0.850	4.700	0.990	0.980	0.920	1.000	1.000	0.172	0.116	0.270	0.640	0.360	0.270	0.063	0.000	0.000	0.000	0.000
Aquicella	0.000	0.000	0.003	11.000	0.890	0.990	0.840	1.000	1.000	0.170	0.941	0.563	0.058	0.008	0.123	0.012	0.000	0.000	0.000	0.000
Amycolatopsis	0.000	0.000	0.000	22.000	0.970	0.500	0.870	1.000	1.000	0.169	0.000	0.014	0.504	0.884	0.090	0.203	0.000	0.000	0.000	0.000
Ellin6067	0.003	0.011	1.000	4.500	0.740	0.970	1.000	1.000	1.000	0.147	0.314	0.922	0.203	0.036	0.000	0.000	0.000	0.000	0.000	0.000
Bryobacter	0.006	0.021	1.000	3.800	0.044	1.000	1.000	1.000	1.000	0.145	0.017	1.020	0.260	0.152	0.000	0.000	0.000	0.000	0.000	0.000
RB41	0.000	0.000	0.000	22.000	0.450	0.980	1.000	1.000	1.000	0.144	0.846	0.490	0.078	0.029	0.000	0.000	0.000	0.000	0.000	0.000
Mucilaginibacter	0.000	0.000	0.000	41.000	0.530	0.420	1.000	1.000	1.000	0.130	0.000	0.026	0.774	0.504	0.000	0.000	0.000	0.000	0.000	0.000
Acidibacter	0.000	0.000	0.002	11.000	0.000	0.600	1.000	0.930	1.000	0.126	0.000	0.640	0.436	0.160	0.000	0.000	0.029	0.000	0.000	0.000
Chitinophaga	0.000	0.000	0.000	15.000	1.000	0.098	1.000	1.000	1.000	0.123	0.000	0.002	0.884	0.325	0.014	0.008	0.000	0.000	0.000	0.000
HSB_OF53F07	0.020	0.052	1.000	3.000	0.120	1.000	1.000	1.000	1.000	0.118	0.044	1.020	0.090	0.029	0.000	0.000	0.000	0.000	0.000	0.000
Gemmatimonas	0.000	0.000	0.000	110.000	0.000	0.000	1.000	1.000	1.000	0.116	0.000	0.624	0.423	0.109	0.000	0.000	0.000	0.000	0.000	0.000
MND1	0.007	0.024	1.000	3.700	0.038	0.990	1.000	1.000	1.000	0.115	0.020	0.941	0.152	0.032	0.000	0.000	0.000	0.000	0.000	0.000
Psychrobacter	0.350	0.560	1.000	1.200	0.970	1.000	1.000	0.960	0.870	0.113	0.436	0.073	0.000	0.000	0.006	0.000	0.325	0.026	0.260	0.000
Ramlibacter	0.000	0.000	0.001	13.000	0.860	0.620	1.000	1.000	1.000	0.107	0.314	0.160	0.397	0.185	0.002	0.005	0.003	0.000	0.000	0.000
Halomonas	0.250	0.440	1.000	1.400	0.990	1.000	1.000	0.920	0.880	0.105	0.476	0.152	0.001	0.000	0.000	0.000	0.194	0.000	0.230	0.000
Candidatus_Xiphinematobacter	0.210	0.380	1.000	1.500	1.000	1.000	0.980	0.660	1.000	0.103	0.000	0.006	0.003	0.000	0.518	0.130	0.372	0.000	0.000	0.000
uncultured_Chloroflexi_bacterium	0.000	0.000	0.000	19.000	0.200	0.980	1.000	1.000	1.000	0.099	0.548	0.260	0.096	0.048	0.026	0.008	0.000	0.000	0.000	0.000
Pseudonocardia	0.000	0.000	0.000	68.000	0.180	0.990	0.990	1.000	1.000	0.096	0.000	0.020	0.504	0.436	0.000	0.003	0.000	0.000	0.000	0.000
Rhodanobacter	0.000	0.000	0.001	12.000	0.000	1.000	1.000	1.000	1.000	0.095	0.000	0.608	0.152	0.194	0.000	0.000	0.000	0.000	0.000	0.000
SHPL14	0.023	0.057	1.000	2.900	1.000	0.970	0.370	1.000	0.940	0.092	0.048	0.048	0.084	0.005	0.578	0.090	0.000	0.000	0.063	0.000
Variovorax	0.004	0.015	1.000	4.100	1.000	0.990	0.290	1.000	1.000	0.091	0.130	0.096	0.123	0.048	0.423	0.078	0.012	0.000	0.000	0.000
Haliangium	0.021	0.054	1.000	2.900	0.051	0.960	1.000	0.950	1.000	0.085	0.000	0.548	0.194	0.036	0.000	0.000	0.073	0.000	0.000	0.000
Devosia	0.000	0.000	0.000	17.000	1.000	1.000	0.180	1.000	1.000	0.085	0.000	0.000	0.116	0.078	0.194	0.462	0.000	0.000	0.000	0.000
Methylobacterium	0.005	0.019	1.000	3.900	0.850	1.000	1.000	0.560	0.990	0.084	0.314	0.130	0.032	0.032	0.004	0.012	0.250	0.048	0.017	0.000
Mycobacterium	0.340	0.560	1.000	1.200	0.990	1.000	1.000	1.000	1.000	0.083	0.000	0.032	0.168	0.109	0.203	0.160	0.053	0.058	0.020	0.026
uncultured_actinobacterium	0.002	0.010	0.960	4.600	1.000	1.000	1.000	1.000	1.000	0.079	0.360	0.270	0.102	0.058	0.000	0.000	0.000	0.000	0.000	0.000
uncultured_Acidobacteria_bacterium	0.002	0.008	0.730	4.800	0.840	1.000	1.000	1.000	1.000	0.077	0.518	0.221	0.023	0.006	0.000	0.000	0.000	0.000	0.000	0.000
Gemmata	0.008	0.026	1.000	3.600	1.000	0.730	1.000	1.000	1.000	0.072	0.152	0.160	0.270	0.078	0.029	0.003	0.005	0.020	0.000	0.000
metagenome	0.006	0.022	1.000	3.800	0.053	0.900	0.900	1.000	0.710	0.070	0.000	0.281	0.221	0.068	0.048	0.000	0.000	0.000	0.078	0.000
Acinetobacter	0.130	0.250	1.000	1.800	1.000	1.000	1.000	0.750	1.000	0.069	0.212	0.123	0.044	0.048	0.006	0.000	0.023	0.221	0.000	0.017

	One-way ANOVA				P values of Tukey's HSD post hoc					Average relative abundance among plant compartments and fertilizer treatments (C: control, N: fertilized)											
Taxa	P Group	FDR Group	P adjusted Group	F Value Group	BS	Rh	R	Stem	Leaf	Average	BS-C mean	BS-N mean	Rh-C mean	Rh-N mean	R-C mean	R-N mean	Stem-C mean	Stem-N mean	Leaf-C mean	Leaf-N mean
Coxiella	0.003	0.011	1.000	4.500	0.570	1.000	1.000	1.000	1.000	0.069	0.518	0.152	0.017	0.002	0.001	0.001	0.001	0.000	0.000	0.000
Candidatus_Solibacter	0.004	0.015	1.000	4.200	0.009	1.000	1.000	1.000	1.000	0.068	0.000	0.518	0.123	0.040	0.000	0.000	0.000	0.000	0.000	0.000
Taibaiella	0.000	0.000	0.000	22.000	1.000	0.980	0.150	1.000	1.000	0.068	0.000	0.000	0.168	0.116	0.116	0.281	0.000	0.000	0.000	0.000
uncultured_soil_bacterium	0.010	0.029	1.000	3.500	0.160	0.900	1.000	0.980	1.000	0.065	0.010	0.314	0.230	0.063	0.003	0.000	0.000	0.032	0.000	0.000
Candidatus_Koribacter	0.002	0.009	0.760	4.700	0.044	1.000	1.000	1.000	1.000	0.065	0.029	0.490	0.090	0.036	0.000	0.000	0.000	0.000	0.000	0.000
Candidatus_Protochlamydia	0.025	0.061	1.000	2.800	1.000	1.000	0.090	1.000	1.000	0.063	0.008	0.017	0.029	0.001	0.548	0.020	0.009	0.000	0.000	0.000
Candidatus_Udaeobacter	0.000	0.000	0.001	13.000	0.034	0.830	1.000	1.000	1.000	0.060	0.109	0.423	0.058	0.009	0.000	0.000	0.000	0.000	0.000	0.000
ADurb.Bin0631	0.006	0.022	1.000	3.800	0.011	1.000	1.000	1.000	1.000	0.059	0.000	0.490	0.073	0.026	0.000	0.000	0.000	0.000	0.000	0.000
Pandanus_utilis	0.110	0.210	1.000	1.900	1.000	1.000	1.000	0.910	0.880	0.059	0.000	0.000	0.000	0.000	0.000	0.000	0.084	0.348	0.152	0.004
Legionella	0.012	0.034	1.000	3.300	1.000	1.000	0.410	1.000	1.000	0.058	0.078	0.029	0.116	0.063	0.260	0.036	0.000	0.000	0.000	0.000
Tumebacillus	0.360	0.560	1.000	1.200	1.000	1.000	1.000	1.000	0.940	0.057	0.000	0.000	0.000	0.001	0.000	0.000	0.029	0.137	0.348	0.058
Nitrospira	0.001	0.007	0.600	5.000	0.008	1.000	1.000	1.000	1.000	0.057	0.000	0.360	0.109	0.102	0.000	0.000	0.000	0.000	0.000	0.000
Lysobacter	0.000	0.000	0.001	12.000	1.000	0.860	1.000	1.000	1.000	0.057	0.040	0.026	0.303	0.185	0.004	0.012	0.000	0.000	0.000	0.000
Chthoniobacter	0.003	0.014	1.000	4.300	0.640	0.640	0.091	1.000	1.000	0.055	0.000	0.068	0.185	0.029	0.260	0.006	0.000	0.000	0.000	0.000
Cutibacterium	0.540	0.570	1.000	0.910	1.000	1.000	1.000	1.000	0.950	0.055	0.053	0.026	0.000	0.000	0.001	0.000	0.036	0.109	0.281	0.040
Flavisolibacter	0.000	0.000	0.005	10.000	1.000	1.000	1.000	1.000	1.000	0.052	0.026	0.007	0.221	0.270	0.000	0.000	0.000	0.000	0.000	0.000
Lactobacillus	0.540	0.570	1.000	0.910	1.000	1.000	1.000	0.990	0.650	0.051	0.036	0.002	0.001	0.000	0.000	0.000	0.090	0.000	0.384	0.002
Hyphomicrobium	0.003	0.014	1.000	4.200	0.013	1.000	1.000	1.000	1.000	0.051	0.000	0.260	0.102	0.063	0.048	0.040	0.000	0.000	0.000	0.000
Luteolibacter	0.004	0.017	1.000	4.100	1.000	0.930	0.310	1.000	1.000	0.051	0.000	0.000	0.058	0.002	0.384	0.068	0.000	0.000	0.000	0.000
Rudaea	0.000	0.000	0.003	11.000	0.280	0.003	1.000	1.000	1.000	0.051	0.000	0.053	0.410	0.044	0.000	0.000	0.000	0.000	0.000	0.000
Pajaroellobacter	0.003	0.012	1.000	4.400	0.130	0.850	0.870	0.980	1.000	0.046	0.004	0.168	0.185	0.063	0.029	0.000	0.017	0.000	0.000	0.000
Nonomuraea	0.000	0.000	0.000	25.000	1.000	0.770	0.990	1.000	1.000	0.046	0.000	0.000	0.137	0.212	0.068	0.044	0.000	0.000	0.000	0.000
Terrimicrobium	0.044	0.098	1.000	2.500	0.990	0.680	0.280	1.000	1.000	0.046	0.029	0.000	0.109	0.000	0.303	0.014	0.000	0.000	0.000	0.002
Sinomonas	0.000	0.000	0.002	11.000	0.840	0.001	0.930	1.000	1.000	0.045	0.000	0.017	0.029	0.397	0.000	0.012	0.000	0.000	0.000	0.000
Bdellovibrio	0.001	0.004	0.300	5.600	0.600	0.440	0.390	1.000	1.000	0.045	0.000	0.044	0.221	0.058	0.116	0.010	0.000	0.000	0.000	0.000
uncultured_Firmicutes_bacterium	0.003	0.012	1.000	4.400	0.870	1.000	1.000	1.000	1.000	0.043	0.109	0.270	0.036	0.017	0.000	0.000	0.000	0.000	0.000	0.000
Brevibacterium	0.240	0.430	1.000	1.400	0.980	1.000	1.000	1.000	1.000	0.043	0.250	0.078	0.002	0.000	0.000	0.000	0.063	0.036	0.000	0.000
Dokdonella	0.009	0.027	1.000	3.500	0.790	0.630	1.000	1.000	1.000	0.043	0.020	0.130	0.221	0.048	0.000	0.000	0.010	0.000	0.000	0.000
SWB02	0.000	0.000	0.029	7.900	0.000	0.980	1.000	1.000	1.000	0.043	0.000	0.384	0.036	0.007	0.000	0.000	0.000	0.000	0.000	0.000
Acidobacteria_bacterium_IGE011	0.000	0.000	0.009	9.400	0.032	0.960	1.000	1.000	1.000	0.040	0.303	0.058	0.036	0.008	0.000	0.000	0.000	0.000	0.000	0.000
Pseudoduganella	0.000	0.000	0.000	29.000	0.580	0.000	1.000	1.000	1.000	0.040	0.000	0.010	0.314	0.073	0.000	0.000	0.000	0.000	0.000	0.000
Lacunisphaera	0.000	0.000	0.000	47.000	0.091	0.001	1.000	1.000	1.000	0.038	0.000	0.014	0.260	0.102	0.000	0.000	0.000	0.000	0.000	0.000
EscherichiaShigella	0.040	0.091	1.000	2.500	0.530	1.000	1.000	1.000	0.950	0.037	0.270	0.036	0.000	0.000	0.000	0.000	0.000	0.000	0.063	0.003
Lysinibacillus	0.027	0.064	1.000	2.800	0.420	1.000	1.000	1.000	1.000	0.037	0.303	0.044	0.001	0.000	0.000	0.000	0.009	0.000	0.000	0.012

	One-way ANOVA				P values of Tukey's HSD post hoc					Average relative abundance among plant compartments and fertilizer treatments (C: control, N: fertilized)										
Taxa	P Group	FDR Group	P adjusted Group	F Value Group	BS	Rh	R	Stem	Leaf	Average	BS-C mean	BS-N mean	Rh-C mean	Rh-N mean	R-C mean	R-N mean	Stem-C mean	Stem-N mean	Leaf-C mean	Leaf-N mean
uncultured_Armatimonadetes_bacterium	0.003	0.014	1.000	4.300	0.011	0.980	1.000	1.000	1.000	0.037	0.000	0.260	0.084	0.023	0.000	0.000	0.000	0.000	0.000	0.000
Prosthecobacter	0.012	0.034	1.000	3.300	1.000	0.980	0.017	1.000	1.000	0.036	0.000	0.000	0.023	0.000	0.336	0.000	0.000	0.000	0.000	0.000
Nordella	0.016	0.043	1.000	3.100	0.910	0.980	1.000	1.000	1.000	0.036	0.053	0.152	0.102	0.036	0.014	0.001	0.000	0.000	0.000	0.000
Candidatus_Ovatusbacter	0.025	0.061	1.000	2.800	0.990	1.000	1.000	1.000	1.000	0.036	0.116	0.230	0.010	0.000	0.001	0.000	0.000	0.000	0.000	0.000
Chthonomonas	0.003	0.013	1.000	4.300	0.011	0.970	1.000	1.000	1.000	0.034	0.000	0.240	0.078	0.023	0.000	0.000	0.000	0.000	0.000	0.000
Vicinamibacter	0.041	0.093	1.000	2.500	0.900	0.980	0.990	1.000	1.000	0.034	0.036	0.137	0.102	0.032	0.029	0.004	0.000	0.000	0.000	0.000
uncultured_microorganism	0.017	0.045	1.000	3.100	1.000	0.046	1.000	1.000	1.000	0.034	0.000	0.000	0.336	0.003	0.000	0.000	0.000	0.000	0.000	0.000
Microbacterium	0.008	0.026	1.000	3.600	0.960	1.000	0.430	1.000	1.000	0.034	0.020	0.000	0.026	0.029	0.044	0.221	0.000	0.000	0.000	0.000
Nitrosospira	0.000	0.001	0.050	7.300	0.000	0.640	1.000	1.000	1.000	0.033	0.000	0.270	0.004	0.058	0.000	0.000	0.000	0.000	0.000	0.000
Pirellula	0.000	0.000	0.022	8.300	0.010	0.420	0.980	1.000	1.000	0.032	0.000	0.102	0.160	0.048	0.012	0.001	0.000	0.000	0.000	0.000
Listeria	0.240	0.430	1.000	1.400	1.000	1.000	1.000	1.000	0.440	0.032	0.040	0.102	0.000	0.000	0.000	0.000	0.000	0.009	0.168	0.000
Pseudorhodoplanes	0.600	0.610	1.000	0.830	1.000	1.000	1.000	1.000	1.000	0.031	0.010	0.000	0.023	0.001	0.003	0.000	0.029	0.044	0.053	0.152
Phenylobacterium	0.000	0.000	0.000	23.000	0.031	0.036	1.000	1.000	1.000	0.031	0.000	0.032	0.203	0.078	0.001	0.000	0.000	0.000	0.000	0.000
Brevibacillus	0.520	0.570	1.000	0.930	1.000	1.000	0.530	1.000	1.000	0.031	0.000	0.000	0.000	0.000	0.281	0.000	0.000	0.032	0.000	0.000
Pseudoxanthomonas	0.003	0.013	1.000	4.300	1.000	0.990	0.980	1.000	1.000	0.030	0.000	0.000	0.096	0.044	0.053	0.109	0.000	0.000	0.000	0.000
Candidatus_Rubidus	0.000	0.000	0.008	9.600	1.000	1.000	0.990	1.000	1.000	0.028	0.000	0.000	0.001	0.002	0.168	0.109	0.000	0.000	0.000	0.000
OLB17	0.000	0.001	0.077	6.900	0.068	0.420	1.000	1.000	1.000	0.028	0.000	0.073	0.160	0.044	0.000	0.000	0.000	0.000	0.000	0.000
Macrococcus	0.029	0.069	1.000	2.700	0.032	1.000	1.000	1.000	1.000	0.027	0.240	0.000	0.000	0.004	0.000	0.000	0.006	0.017	0.000	0.000
Piscinibacter	0.000	0.000	0.026	8.000	0.980	0.035	1.000	1.000	1.000	0.026	0.000	0.007	0.221	0.036	0.000	0.000	0.000	0.000	0.000	0.000
Neochlamydia	0.000	0.003	0.210	5.900	0.930	0.930	0.960	1.000	1.000	0.026	0.004	0.026	0.044	0.012	0.116	0.063	0.000	0.000	0.000	0.000
Aeromicrobium	0.000	0.000	0.000	14.000	1.000	0.061	0.920	1.000	1.000	0.026	0.000	0.001	0.194	0.063	0.006	0.000	0.000	0.000	0.000	0.000
X19213	0.013	0.037	1.000	3.300	0.022	1.000	1.000	1.000	1.000	0.026	0.000	0.212	0.036	0.009	0.000	0.000	0.000	0.000	0.000	0.000
Azospirillum	0.000	0.000	0.001	13.000	1.000	1.000	1.000	1.000	1.000	0.025	0.000	0.000	0.116	0.137	0.000	0.000	0.000	0.000	0.000	0.000
mle17	0.010	0.029	1.000	3.400	0.046	0.990	1.000	1.000	1.000	0.025	0.005	0.230	0.012	0.000	0.000	0.000	0.000	0.000	0.000	0.000
Rhodoplanes	0.000	0.000	0.000	22.000	0.000	0.990	0.720	1.000	1.000	0.024	0.000	0.160	0.044	0.029	0.001	0.009	0.000	0.000	0.000	0.000
MM2	0.000	0.000	0.000	73.000	1.000	0.058	1.000	1.000	1.000	0.022	0.000	0.000	0.137	0.084	0.000	0.000	0.000	0.000	0.000	0.000
Enterococcus	0.082	0.160	1.000	2.100	0.530	1.000	1.000	0.460	1.000	0.021	0.000	0.068	0.001	0.002	0.000	0.000	0.008	0.130	0.000	0.007
Candidatus_Berkiella	0.004	0.015	1.000	4.100	1.000	1.000	0.300	1.000	1.000	0.021	0.000	0.003	0.017	0.006	0.152	0.029	0.000	0.000	0.000	0.000
Polycyclovorans	0.004	0.015	1.000	4.200	0.019	0.970	1.000	1.000	1.000	0.020	0.000	0.130	0.058	0.017	0.000	0.000	0.000	0.000	0.000	0.000
Methylophilus	0.000	0.000	0.031	7.900	0.012	0.960	1.000	1.000	1.000	0.020	0.000	0.068	0.084	0.044	0.000	0.000	0.000	0.000	0.000	0.000
Acidothermus	0.022	0.055	1.000	2.900	0.270	0.950	1.000	1.000	1.000	0.019	0.014	0.152	0.023	0.001	0.000	0.000	0.000	0.000	0.000	0.000
Cohnella	0.440	0.560	1.000	1.000	1.000	1.000	0.980	1.000	0.540	0.018	0.000	0.002	0.012	0.000	0.048	0.002	0.000	0.000	0.116	0.000
Bordetella	0.001	0.003	0.240	5.800	1.000	1.000	0.150	1.000	1.000	0.018	0.000	0.002	0.014	0.008	0.130	0.026	0.000	0.000	0.000	0.000
Gordonia	0.000	0.000	0.000	30.000	1.000	0.540	1.000	1.000	1.000	0.017	0.000	0.000	0.044	0.073	0.029	0.029	0.000	0.000	0.000	0.000

	One-way ANOVA				P values of Tukey’s HSD post hoc					Average relative abundance among plant compartments and fertilizer treatments (C: control, N: fertilized)										
Taxa	P Group	FDR Group	P adjusted Group	F Value Group	BS	Rh	R	Stem	Leaf	Average	BS-C mean	BS-N mean	Rh-C mean	Rh-N mean	R-C mean	R-N mean	Stem-C mean	Stem-N mean	Leaf-C mean	Leaf-N mean
uncultured_delta_proteobacterium	0.001	0.003	0.270	5.700	0.021	0.510	1.000	1.000	1.000	0.017	0.000	0.078	0.078	0.017	0.000	0.000	0.000	0.000	0.000	0.000
Nocardia	0.000	0.002	0.130	6.400	1.000	0.920	1.000	1.000	1.000	0.017	0.000	0.002	0.001	0.014	0.090	0.063	0.000	0.000	0.000	0.000
Pseudoflavitalea	0.002	0.010	0.930	4.600	0.220	0.550	1.000	1.000	1.000	0.016	0.000	0.044	0.096	0.023	0.000	0.000	0.000	0.000	0.000	0.000
Vagococcus	0.200	0.370	1.000	1.500	1.000	1.000	1.000	0.370	1.000	0.016	0.032	0.012	0.000	0.000	0.000	0.000	0.116	0.001	0.000	0.000
UTBCD1	0.011	0.032	1.000	3.400	0.013	1.000	1.000	1.000	1.000	0.016	0.000	0.152	0.004	0.004	0.000	0.000	0.000	0.000	0.000	0.000
Patulibacter	0.000	0.000	0.028	8.000	0.089	0.840	1.000	1.000	1.000	0.016	0.032	0.000	0.084	0.040	0.001	0.001	0.000	0.000	0.000	0.000
Enhydrobacter	0.083	0.160	1.000	2.100	0.093	1.000	1.000	0.970	1.000	0.016	0.130	0.000	0.000	0.000	0.004	0.000	0.000	0.017	0.000	0.006
Iamia	0.031	0.072	1.000	2.700	0.068	1.000	1.000	1.000	1.000	0.016	0.000	0.102	0.036	0.014	0.002	0.000	0.000	0.000	0.000	0.000
Actinoallomurus	0.000	0.000	0.000	14.000	1.000	0.110	1.000	1.000	1.000	0.015	0.000	0.000	0.036	0.096	0.012	0.008	0.000	0.000	0.000	0.000
Actinomycetospora	0.000	0.001	0.059	7.200	1.000	0.610	1.000	0.039	1.000	0.015	0.000	0.000	0.078	0.029	0.000	0.000	0.044	0.000	0.000	0.000
Anaerococcus	0.530	0.570	1.000	0.920	1.000	1.000	1.000	1.000	0.710	0.015	0.000	0.000	0.000	0.000	0.000	0.000	0.003	0.000	0.144	0.004
Paracoccus	0.470	0.560	1.000	1.000	1.000	1.000	1.000	1.000	0.470	0.014	0.000	0.000	0.000	0.000	0.000	0.000	0.000	0.000	0.144	0.000
Luteibacter	0.000	0.000	0.001	13.000	1.000	0.001	0.990	1.000	1.000	0.014	0.000	0.000	0.130	0.012	0.002	0.000	0.000	0.000	0.000	0.000
Xanthomonas	0.009	0.027	1.000	3.600	1.000	0.990	0.320	0.990	1.000	0.014	0.000	0.000	0.036	0.014	0.009	0.078	0.000	0.004	0.000	0.000
uncultured_Syntrophobacterales_bacterium	0.000	0.000	0.000	30.000	0.000	0.800	1.000	1.000	1.000	0.014	0.000	0.137	0.003	0.000	0.000	0.000	0.000	0.000	0.000	0.000
Gemmatirosa	0.000	0.000	0.000	28.000	0.000	0.650	1.000	1.000	1.000	0.014	0.000	0.116	0.017	0.006	0.000	0.000	0.000	0.000	0.000	0.000
Fimbriiglobus	0.000	0.000	0.009	9.400	0.140	0.120	0.410	1.000	1.000	0.014	0.000	0.017	0.078	0.020	0.020	0.001	0.000	0.000	0.000	0.000
uncultured_proteobacterium	0.000	0.001	0.094	6.700	0.920	0.034	0.870	1.000	1.000	0.014	0.004	0.020	0.090	0.012	0.009	0.000	0.000	0.000	0.000	0.000
Flavitalea	0.000	0.000	0.006	10.000	0.082	0.990	1.000	1.000	1.000	0.013	0.000	0.023	0.068	0.044	0.000	0.000	0.000	0.000	0.000	0.000
Actinomadura	0.002	0.008	0.640	4.900	1.000	0.970	0.920	1.000	1.000	0.013	0.000	0.002	0.029	0.058	0.032	0.010	0.000	0.000	0.000	0.000
Conexibacter	0.000	0.000	0.027	8.000	0.170	1.000	1.000	1.000	1.000	0.012	0.000	0.017	0.053	0.040	0.006	0.007	0.000	0.000	0.000	0.000
uncultured_Acidobacteriaceae_bacterium	0.010	0.029	1.000	3.500	0.480	1.000	1.000	1.000	1.000	0.012	0.078	0.014	0.017	0.012	0.000	0.000	0.000	0.000	0.000	0.000
Arcticibacter	0.000	0.000	0.002	11.000	1.000	0.010	1.000	1.000	1.000	0.012	0.000	0.000	0.102	0.017	0.000	0.000	0.000	0.000	0.000	0.000
Methylobacillus	0.000	0.000	0.001	13.000	0.920	0.990	1.000	1.000	1.000	0.012	0.000	0.003	0.048	0.068	0.000	0.000	0.000	0.000	0.000	0.000
Pir4_lineage	0.026	0.063	1.000	2.800	0.200	0.800	0.970	1.000	1.000	0.012	0.000	0.048	0.053	0.009	0.008	0.000	0.000	0.000	0.000	0.000
Leuconostoc	0.470	0.560	1.000	1.000	1.000	1.000	1.000	0.470	1.000	0.012	0.000	0.000	0.000	0.000	0.000	0.000	0.116	0.000	0.000	0.000
Kouleothrix	0.007	0.024	1.000	3.700	0.060	0.970	1.000	1.000	1.000	0.011	0.000	0.058	0.040	0.014	0.000	0.000	0.000	0.000	0.000	0.000
Clostridium_sensu_stricto_1	0.093	0.180	1.000	2.000	1.000	1.000	0.220	1.000	1.000	0.011	0.004	0.000	0.007	0.008	0.090	0.003	0.000	0.000	0.000	0.000
CL50029_marine_group	0.000	0.000	0.005	10.000	0.810	0.600	0.330	1.000	1.000	0.011	0.000	0.004	0.040	0.014	0.040	0.012	0.000	0.000	0.000	0.000
uncultured_Prosthecobacter_sp.	0.008	0.026	1.000	3.600	0.014	1.000	1.000	1.000	1.000	0.011	0.000	0.090	0.014	0.005	0.000	0.000	0.000	0.000	0.000	0.000
Nevskia	0.000	0.000	0.010	9.200	0.460	0.082	1.000	1.000	1.000	0.011	0.000	0.010	0.078	0.020	0.000	0.000	0.000	0.000	0.000	0.000
Massilia	0.002	0.010	0.960	4.500	0.740	0.680	1.000	1.000	1.000	0.010	0.000	0.012	0.068	0.023	0.000	0.000	0.000	0.000	0.000	0.000

	One-way ANOVA				P values of Tukey’s HSD post hoc					Average relative abundance among plant compartments and fertilizer treatments (C: control, N: fertilized)										
Taxa	P Group	FDR Group	P adjusted Group	F Value Group	BS	Rh	R	Stem	Leaf	Average	BS-C mean	BS-N mean	Rh-C mean	Rh-N mean	R-C mean	R-N mean	Stem-C mean	Stem-N mean	Leaf-C mean	Leaf-N mean
Rubrobacter	0.560	0.580	1.000	0.870	0.970	1.000	1.000	1.000	1.000	0.010	0.048	0.008	0.017	0.012	0.000	0.000	0.009	0.000	0.000	0.002
Ruminococcus_2	0.078	0.160	1.000	2.100	0.110	1.000	1.000	1.000	1.000	0.009	0.000	0.073	0.014	0.006	0.001	0.000	0.000	0.000	0.000	0.000
uncultured_Nitrosomonadaceae_bacterium	0.025	0.061	1.000	2.800	0.034	1.000	1.000	1.000	1.000	0.009	0.000	0.078	0.006	0.006	0.000	0.000	0.000	0.000	0.000	0.000
Microlunatus	0.000	0.001	0.044	7.500	1.000	0.200	0.290	1.000	1.000	0.009	0.000	0.000	0.010	0.048	0.003	0.029	0.000	0.000	0.000	0.000
FCPS473	0.007	0.024	1.000	3.700	0.009	1.000	1.000	1.000	1.000	0.009	0.000	0.090	0.000	0.000	0.000	0.000	0.000	0.000	0.000	0.000
Asticcacaulis	0.000	0.000	0.000	20.000	1.000	0.850	1.000	1.000	1.000	0.009	0.000	0.000	0.053	0.036	0.000	0.000	0.000	0.000	0.000	0.000
Edaphobacter	0.000	0.000	0.007	9.700	1.000	1.000	0.770	1.000	1.000	0.009	0.000	0.000	0.044	0.040	0.000	0.004	0.000	0.000	0.000	0.000
unidentified	0.044	0.098	1.000	2.500	0.990	1.000	1.000	1.000	1.000	0.009	0.026	0.058	0.004	0.000	0.000	0.000	0.000	0.000	0.000	0.000
Holophaga	0.000	0.002	0.150	6.200	0.590	0.990	1.000	1.000	1.000	0.009	0.009	0.000	0.048	0.029	0.000	0.000	0.000	0.000	0.000	0.000
Asinibacterium	0.000	0.000	0.032	7.800	0.960	0.550	1.000	1.000	1.000	0.008	0.000	0.003	0.058	0.023	0.000	0.000	0.000	0.000	0.000	0.000
Pedomicrobium	0.033	0.076	1.000	2.700	0.038	1.000	1.000	1.000	1.000	0.008	0.000	0.073	0.006	0.001	0.000	0.000	0.000	0.000	0.000	0.000
Domibacillus	0.560	0.580	1.000	0.880	1.000	1.000	1.000	0.980	0.640	0.008	0.000	0.000	0.000	0.001	0.000	0.000	0.017	0.000	0.063	0.000
X19212	0.009	0.027	1.000	3.600	0.011	1.000	1.000	1.000	1.000	0.008	0.000	0.078	0.001	0.001	0.000	0.000	0.000	0.000	0.000	0.000
uncultured_Myxococcales_bacterium	0.350	0.560	1.000	1.200	1.000	1.000	1.000	1.000	1.000	0.008	0.040	0.036	0.001	0.001	0.000	0.000	0.000	0.000	0.000	0.000
Phaseolus_acutifolius_tepary_bean	0.470	0.560	1.000	1.000	0.470	1.000	1.000	1.000	1.000	0.008	0.078	0.000	0.000	0.000	0.000	0.000	0.000	0.000	0.000	0.000
Thermosporothrix	0.000	0.002	0.150	6.200	0.130	0.990	0.970	1.000	1.000	0.008	0.000	0.017	0.036	0.023	0.003	0.000	0.000	0.000	0.000	0.000
Noviherbaspirillum	0.000	0.000	0.000	15.000	1.000	1.000	1.000	1.000	1.000	0.008	0.000	0.000	0.044	0.032	0.000	0.000	0.000	0.000	0.000	0.000
Renibacterium	0.027	0.064	1.000	2.800	0.040	1.000	1.000	0.940	1.000	0.008	0.068	0.000	0.000	0.000	0.000	0.000	0.009	0.000	0.000	0.000
uncultured_Caldilineae_bacterium	0.250	0.440	1.000	1.400	1.000	1.000	1.000	1.000	1.000	0.008	0.040	0.036	0.000	0.000	0.000	0.000	0.000	0.000	0.000	0.000
Roseimicrobium	0.024	0.060	1.000	2.900	0.930	0.850	0.400	1.000	1.000	0.008	0.000	0.006	0.029	0.006	0.032	0.001	0.000	0.000	0.000	0.000
uncultured_Geothrix_sp.	0.016	0.043	1.000	3.100	0.056	1.000	1.000	1.000	1.000	0.008	0.000	0.048	0.017	0.010	0.000	0.000	0.000	0.000	0.000	0.000
Streptococcus	0.070	0.140	1.000	2.200	1.000	1.000	1.000	1.000	1.000	0.008	0.040	0.032	0.000	0.000	0.000	0.000	0.003	0.000	0.000	0.000
Actinocorallia	0.000	0.000	0.014	8.800	0.600	0.490	1.000	1.000	1.000	0.007	0.000	0.005	0.048	0.020	0.000	0.000	0.000	0.000	0.000	0.000
Kibdelosporangium	0.000	0.000	0.007	9.700	1.000	0.250	0.380	1.000	1.000	0.007	0.000	0.000	0.048	0.017	0.000	0.007	0.000	0.000	0.000	0.000
uncultured_Anaeromyxobacter_sp.	0.210	0.380	1.000	1.500	1.000	1.000	1.000	1.000	1.000	0.007	0.026	0.044	0.002	0.000	0.000	0.000	0.000	0.000	0.000	0.000
uncultured_Acetobacteraceae_bacterium	0.000	0.002	0.110	6.600	0.210	0.800	1.000	1.000	1.000	0.007	0.000	0.014	0.040	0.017	0.000	0.000	0.000	0.000	0.000	0.000
Parasegetibacter	0.000	0.000	0.000	19.000	1.000	0.190	1.000	1.000	1.000	0.007	0.000	0.000	0.048	0.023	0.000	0.000	0.000	0.000	0.000	0.000
Steroidobacter	0.060	0.130	1.000	2.300	0.780	0.110	1.000	1.000	1.000	0.007	0.000	0.017	0.053	0.000	0.000	0.000	0.000	0.000	0.000	0.000
uncultured_forest_soil_bacterium	0.009	0.027	1.000	3.600	0.024	0.980	1.000	1.000	1.000	0.007	0.000	0.048	0.017	0.004	0.000	0.000	0.000	0.000	0.000	0.000

	One-way ANOVA				P values of Tukey's HSD post hoc					Average relative abundance among plant compartments and fertilizer treatments (C: control, N: fertilized)										
Taxa	P Group	FDR Group	P adjusted Group	F Value Group	BS	Rh	R	Stem	Leaf	Average	BS-C mean	BS-N mean	Rh-C mean	Rh-N mean	R-C mean	R-N mean	Stem-C mean	Stem-N mean	Leaf-C mean	Leaf-N mean
Dactylosporangium	0.100	0.190	1.000	1.900	0.930	1.000	1.000	1.000	1.000	0.007	0.012	0.040	0.012	0.004	0.000	0.000	0.000	0.000	0.000	0.000
Pseudoalteromonas	0.640	0.650	1.000	0.780	0.950	1.000	1.000	0.980	1.000	0.007	0.044	0.004	0.000	0.000	0.000	0.000	0.017	0.000	0.002	0.000
uncultured_organism	0.027	0.064	1.000	2.800	0.046	0.990	1.000	1.000	1.000	0.007	0.000	0.053	0.012	0.002	0.000	0.000	0.000	0.000	0.000	0.000
SM1A02	0.014	0.039	1.000	3.200	0.170	0.410	1.000	1.000	1.000	0.007	0.000	0.029	0.036	0.003	0.000	0.000	0.000	0.000	0.000	0.000
Sphingobacterium	0.190	0.350	1.000	1.600	1.000	1.000	0.670	1.000	1.000	0.007	0.000	0.000	0.014	0.023	0.000	0.029	0.000	0.000	0.000	0.000
uncultured_Holophaga_sp.	0.140	0.270	1.000	1.800	0.150	1.000	1.000	1.000	1.000	0.006	0.000	0.063	0.000	0.000	0.000	0.000	0.000	0.000	0.000	0.000
Terrimonas	0.002	0.010	0.960	4.600	0.660	0.012	1.000	1.000	1.000	0.006	0.000	0.009	0.053	0.000	0.000	0.000	0.000	0.000	0.000	0.000
Sporichthya	0.095	0.190	1.000	2.000	0.490	1.000	1.000	1.000	1.000	0.006	0.023	0.000	0.023	0.017	0.000	0.000	0.000	0.000	0.000	0.000
Mitsuaria	0.031	0.072	1.000	2.700	1.000	0.036	1.000	1.000	1.000	0.006	0.000	0.000	0.058	0.000	0.001	0.000	0.000	0.000	0.000	0.000
uncultured_alpha_proteobacterium	0.025	0.061	1.000	2.800	0.031	1.000	1.000	1.000	1.000	0.006	0.058	0.000	0.000	0.000	0.000	0.000	0.000	0.000	0.000	0.000
Pelomonas	0.001	0.003	0.250	5.700	0.250	0.014	1.000	1.000	1.000	0.006	0.000	0.012	0.044	0.001	0.000	0.000	0.000	0.000	0.000	0.000
Pedosphaera	0.045	0.100	1.000	2.500	0.058	0.990	1.000	1.000	1.000	0.006	0.000	0.053	0.004	0.000	0.000	0.000	0.000	0.000	0.000	0.000
Dyadobacter	0.000	0.000	0.016	8.600	1.000	0.560	1.000	1.000	1.000	0.006	0.000	0.000	0.040	0.017	0.000	0.000	0.000	0.000	0.000	0.000
Niabella	0.000	0.000	0.000	16.000	1.000	0.790	0.980	1.000	1.000	0.006	0.000	0.000	0.020	0.032	0.001	0.003	0.000	0.000	0.000	0.000
Rivibacter	0.005	0.018	1.000	4.000	1.000	1.000	1.000	1.000	1.000	0.005	0.000	0.000	0.032	0.023	0.000	0.000	0.000	0.000	0.000	0.000
Singulisphaera	0.082	0.160	1.000	2.100	0.130	0.970	1.000	1.000	1.000	0.005	0.000	0.036	0.014	0.002	0.001	0.001	0.000	0.000	0.000	0.000
Minicystis	0.022	0.055	1.000	2.900	0.046	0.980	1.000	1.000	1.000	0.005	0.000	0.040	0.012	0.002	0.000	0.000	0.000	0.000	0.000	0.000
Arcobacter	0.490	0.570	1.000	0.970	1.000	1.000	1.000	0.480	1.000	0.005	0.000	0.000	0.000	0.000	0.001	0.000	0.053	0.000	0.000	0.000
uncultured_bacterium_259	0.470	0.560	1.000	1.000	0.480	1.000	1.000	1.000	1.000	0.005	0.048	0.000	0.005	0.000	0.000	0.000	0.000	0.000	0.000	0.000
Marinilactibacillus	0.480	0.570	1.000	0.980	1.000	1.000	1.000	0.470	1.000	0.005	0.000	0.000	0.000	0.000	0.000	0.000	0.053	0.000	0.000	0.000
Rosenbergiella	0.470	0.560	1.000	1.000	0.470	1.000	1.000	1.000	1.000	0.005	0.053	0.000	0.000	0.000	0.000	0.000	0.000	0.000	0.000	0.000
JG30aKF32	0.099	0.190	1.000	2.000	0.110	1.000	1.000	1.000	1.000	0.005	0.000	0.048	0.003	0.001	0.000	0.000	0.000	0.000	0.000	0.000
Verruc01	0.000	0.000	0.007	9.700	1.000	0.970	0.300	1.000	1.000	0.005	0.000	0.000	0.005	0.002	0.032	0.012	0.000	0.000	0.000	0.000
uncultured_subdivision_3_bacterium	0.000	0.000	0.000	21.000	0.950	0.069	1.000	1.000	1.000	0.005	0.000	0.001	0.036	0.014	0.000	0.000	0.000	0.000	0.000	0.000
AAP99	0.016	0.043	1.000	3.100	1.000	1.000	0.042	1.000	1.000	0.005	0.000	0.000	0.002	0.000	0.048	0.000	0.000	0.000	0.000	0.000
Schlesneria	0.002	0.009	0.760	4.700	0.990	0.440	0.200	1.000	1.000	0.005	0.000	0.002	0.029	0.006	0.012	0.000	0.000	0.000	0.000	0.000
Psychroflexus	0.470	0.560	1.000	1.000	1.000	1.000	1.000	0.470	1.000	0.005	0.000	0.000	0.000	0.000	0.000	0.000	0.048	0.000	0.000	0.000
Diplosphaera	0.022	0.055	1.000	2.900	1.000	0.230	1.000	1.000	1.000	0.005	0.000	0.000	0.044	0.004	0.000	0.000	0.000	0.000	0.000	0.000
Flavobacterium	0.005	0.019	1.000	3.900	0.170	0.530	1.000	1.000	1.000	0.005	0.000	0.017	0.026	0.004	0.000	0.000	0.000	0.000	0.000	0.000
Solirubrobacter	0.065	0.140	1.000	2.200	0.960	0.990	1.000	1.000	1.000	0.005	0.000	0.005	0.029	0.012	0.001	0.000	0.000	0.000	0.000	0.000
Saccharum_hybrid_cultivar	0.200	0.370	1.000	1.600	1.000	1.000	0.390	1.000	1.000	0.005	0.000	0.000	0.000	0.002	0.044	0.001	0.000	0.000	0.000	0.000
uncultured_Gemmatimonadetes_bacterium	0.180	0.330	1.000	1.600	1.000	1.000	1.000	1.000	1.000	0.005	0.017	0.029	0.000	0.001	0.000	0.000	0.000	0.000	0.000	0.000
Ammoniphilus	0.530	0.570	1.000	0.920	1.000	1.000	1.000	0.520	1.000	0.004	0.000	0.004	0.001	0.000	0.000	0.000	0.000	0.040	0.000	0.000

	One-way ANOVA				P values of Tukey’s HSD post hoc					Average relative abundance among plant compartments and fertilizer treatments (C: control, N: fertilized)										
Taxa	P Group	FDR Group	P adjusted Group	F Value Group	BS	Rh	R	Stem	Leaf	Average	BS-C mean	BS-N mean	Rh-C mean	Rh-N mean	R-C mean	R-N mean	Stem-C mean	Stem-N mean	Leaf-C mean	Leaf-N mean
Luedemannella	0.057	0.120	1.000	2.300	0.740	0.940	1.000	1.000	1.000	0.004	0.006	0.032	0.005	0.000	0.000	0.000	0.000	0.000	0.000	0.000
Planctopirus	0.064	0.140	1.000	2.200	0.380	0.490	1.000	0.990	1.000	0.004	0.000	0.017	0.023	0.001	0.000	0.000	0.003	0.000	0.000	0.000
beta_proteobacterium_WW H154	0.025	0.061	1.000	2.800	0.071	0.920	1.000	1.000	1.000	0.004	0.000	0.029	0.012	0.002	0.000	0.000	0.000	0.000	0.000	0.000
Methyloceanibacter	0.011	0.032	1.000	3.400	0.013	1.000	1.000	1.000	1.000	0.004	0.000	0.040	0.001	0.001	0.000	0.000	0.000	0.000	0.000	0.000
uncultured_Conexibacter_sp.	0.020	0.052	1.000	3.000	0.040	0.960	1.000	1.000	1.000	0.004	0.000	0.032	0.008	0.001	0.000	0.000	0.000	0.000	0.000	0.000
Pseudothermotoga	0.059	0.130	1.000	2.300	0.077	0.980	1.000	1.000	1.000	0.004	0.036	0.000	0.004	0.000	0.000	0.000	0.000	0.000	0.000	0.000
IS44	0.022	0.055	1.000	2.900	0.028	1.000	1.000	1.000	1.000	0.004	0.000	0.036	0.003	0.000	0.000	0.000	0.000	0.000	0.000	0.000
Opitutus	0.000	0.000	0.006	9.800	1.000	0.054	1.000	1.000	1.000	0.004	0.000	0.000	0.032	0.007	0.000	0.000	0.000	0.000	0.000	0.000
Novibacillus	0.007	0.025	1.000	3.700	0.013	1.000	1.000	1.000	1.000	0.004	0.000	0.029	0.006	0.004	0.000	0.000	0.000	0.000	0.000	0.000
Geothrix	0.068	0.140	1.000	2.200	0.130	1.000	1.000	1.000	1.000	0.004	0.000	0.026	0.008	0.004	0.000	0.000	0.000	0.000	0.000	0.000
Alkalibacterium	0.620	0.630	1.000	0.810	0.840	1.000	1.000	0.910	1.000	0.004	0.020	0.000	0.000	0.000	0.000	0.000	0.014	0.000	0.000	0.004
Paludibaculum	0.008	0.025	1.000	3.600	0.010	1.000	1.000	1.000	1.000	0.004	0.000	0.036	0.001	0.000	0.000	0.000	0.000	0.000	0.000	0.000
Microvirga	0.000	0.003	0.200	6.000	1.000	1.000	1.000	1.000	1.000	0.004	0.000	0.000	0.020	0.017	0.000	0.000	0.000	0.000	0.000	0.000
Anaeromyxobacter	0.002	0.011	1.000	4.500	0.014	1.000	1.000	1.000	1.000	0.004	0.000	0.023	0.009	0.004	0.000	0.000	0.000	0.000	0.000	0.000
Tetrabaena_socialis	0.470	0.560	1.000	1.000	1.000	0.470	1.000	1.000	1.000	0.004	0.000	0.000	0.000	0.036	0.000	0.000	0.000	0.000	0.000	0.000
Ilumatobacter	0.074	0.150	1.000	2.100	0.078	1.000	1.000	1.000	1.000	0.003	0.000	0.032	0.001	0.001	0.000	0.000	0.000	0.000	0.000	0.000
uncultured_bacterium_gp17	0.500	0.570	1.000	0.960	0.480	1.000	1.000	1.000	1.000	0.003	0.032	0.000	0.001	0.000	0.000	0.000	0.000	0.000	0.000	0.000
Verrucomicrobium	0.490	0.570	1.000	0.980	1.000	0.980	0.900	1.000	1.000	0.003	0.000	0.003	0.017	0.003	0.010	0.000	0.000	0.000	0.000	0.000
uncultured_Acidobacteriales _bacterium	0.002	0.008	0.730	4.800	0.043	0.170	1.000	1.000	1.000	0.003	0.000	0.014	0.017	0.001	0.000	0.000	0.000	0.000	0.000	0.000
Vibrionimonas	0.000	0.000	0.000	21.000	1.000	0.002	1.000	1.000	1.000	0.003	0.000	0.000	0.026	0.006	0.000	0.000	0.000	0.000	0.000	0.000
Lachnoclostridium_5	0.530	0.570	1.000	0.910	1.000	0.600	0.990	1.000	1.000	0.003	0.000	0.000	0.026	0.000	0.006	0.000	0.000	0.000	0.000	0.000
Planifilum	0.120	0.230	1.000	1.900	0.120	1.000	1.000	0.990	0.980	0.003	0.000	0.023	0.002	0.001	0.000	0.000	0.003	0.000	0.003	0.000
Labedaea	0.000	0.000	0.000	17.000	1.000	0.000	1.000	1.000	1.000	0.003	0.000	0.000	0.002	0.029	0.000	0.000	0.000	0.000	0.000	0.000
Subgroup_10	0.003	0.011	1.000	4.500	0.015	0.510	1.000	1.000	1.000	0.003	0.000	0.020	0.010	0.001	0.000	0.000	0.000	0.000	0.000	0.000
Brevundimonas	0.055	0.120	1.000	2.300	1.000	0.200	1.000	1.000	1.000	0.003	0.000	0.000	0.026	0.001	0.003	0.001	0.000	0.000	0.000	0.000
Weissella	0.470	0.560	1.000	1.000	1.000	1.000	1.000	0.470	1.000	0.003	0.000	0.000	0.000	0.000	0.000	0.000	0.000	0.029	0.000	0.000
Vibrio	0.470	0.560	1.000	1.000	1.000	1.000	1.000	0.470	1.000	0.003	0.000	0.000	0.000	0.000	0.000	0.000	0.029	0.000	0.000	0.000
Rubellimicrobium	0.240	0.430	1.000	1.400	0.510	1.000	1.000	1.000	1.000	0.003	0.014	0.000	0.010	0.004	0.000	0.000	0.000	0.000	0.000	0.000
bacterium_Ellin6517	0.009	0.028	1.000	3.500	0.013	0.990	1.000	1.000	1.000	0.003	0.000	0.026	0.002	0.000	0.000	0.000	0.000	0.000	0.000	0.000
LD29	0.460	0.560	1.000	1.000	1.000	0.970	1.000	1.000	1.000	0.003	0.000	0.000	0.020	0.004	0.003	0.000	0.000	0.000	0.000	0.000
Agromyces	0.083	0.160	1.000	2.100	1.000	0.590	0.840	1.000	1.000	0.003	0.000	0.000	0.017	0.001	0.000	0.008	0.000	0.000	0.000	0.000
Chujaibacter	0.000	0.000	0.011	9.100	1.000	0.000	1.000	1.000	1.000	0.003	0.000	0.000	0.000	0.026	0.000	0.000	0.000	0.000	0.000	0.000
Granulicatella	0.470	0.560	1.000	1.000	0.470	1.000	1.000	1.000	1.000	0.003	0.026	0.000	0.000	0.000	0.000	0.000	0.000	0.000	0.000	0.000
Sporomusa	0.520	0.570	1.000	0.940	1.000	0.530	1.000	1.000	1.000	0.002	0.000	0.000	0.023	0.000	0.002	0.000	0.000	0.000	0.000	0.000

	One-way ANOVA				P values of Tukey’s HSD post hoc					Average relative abundance among plant compartments and fertilizer treatments (C: control, N: fertilized)										
Taxa	P Group	FDR Group	P adjusted Group	F Value Group	BS	Rh	R	Stem	Leaf	Average	BS-C mean	BS-N mean	Rh-C mean	Rh-N mean	R-C mean	R-N mean	Stem-C mean	Stem-N mean	Leaf-C mean	Leaf-N mean
Phytophthora_lateralis_MPF4	0.550	0.570	1.000	0.900	1.000	1.000	1.000	1.000	1.000	0.002	0.000	0.000	0.000	0.000	0.000	0.000	0.008	0.017	0.000	0.000
uncultured_Chlamydia_sp.	0.065	0.140	1.000	2.200	0.120	1.000	1.000	1.000	1.000	0.002	0.000	0.017	0.003	0.004	0.000	0.000	0.000	0.000	0.000	0.000
Armatimonadetes_bacterium_5513	0.001	0.003	0.250	5.700	1.000	0.001	0.990	1.000	1.000	0.002	0.000	0.000	0.023	0.000	0.001	0.000	0.000	0.000	0.000	0.000
uncultured_Acidobacterium_sp.	0.016	0.043	1.000	3.100	0.028	0.880	1.000	1.000	1.000	0.002	0.000	0.020	0.003	0.000	0.000	0.000	0.000	0.000	0.000	0.000
Herpetosiphon	0.000	0.000	0.000	20.000	1.000	0.015	1.000	1.000	1.000	0.002	0.000	0.000	0.017	0.005	0.000	0.000	0.000	0.000	0.000	0.000
uncultured_Polyangiaceae_bacterium	0.018	0.047	1.000	3.100	0.580	0.640	1.000	1.000	1.000	0.002	0.000	0.005	0.014	0.003	0.000	0.000	0.000	0.000	0.000	0.000
Corynebacterium_1	0.600	0.610	1.000	0.820	0.810	1.000	1.000	0.920	1.000	0.002	0.012	0.000	0.000	0.000	0.001	0.000	0.008	0.000	0.000	0.000
Jatrophihabitans	0.079	0.160	1.000	2.100	0.890	1.000	1.000	1.000	1.000	0.002	0.000	0.003	0.010	0.007	0.000	0.000	0.000	0.000	0.000	0.000
Blastocatella	0.017	0.045	1.000	3.100	1.000	0.940	1.000	1.000	1.000	0.002	0.000	0.000	0.014	0.005	0.000	0.000	0.000	0.000	0.000	0.000
Sediminibacterium	0.000	0.000	0.000	30.000	1.000	0.670	1.000	1.000	1.000	0.002	0.000	0.000	0.012	0.008	0.000	0.000	0.000	0.000	0.000	0.000
Flavihumibacter	0.470	0.560	1.000	1.000	1.000	0.470	1.000	1.000	1.000	0.002	0.000	0.000	0.020	0.000	0.000	0.000	0.000	0.000	0.000	0.000
uncultured_Sorangiineae_bacterium	0.009	0.027	1.000	3.500	0.045	0.260	1.000	1.000	1.000	0.002	0.000	0.012	0.007	0.000	0.000	0.000	0.000	0.000	0.000	0.000
uncultured_planctomycete	0.053	0.120	1.000	2.400	0.540	0.260	1.000	1.000	1.000	0.002	0.000	0.006	0.012	0.000	0.000	0.000	0.000	0.000	0.000	0.000
Leptolyngbya_EcFYyyy00	0.290	0.510	1.000	1.300	1.000	1.000	1.000	1.000	1.000	0.002	0.000	0.000	0.009	0.010	0.000	0.000	0.000	0.000	0.000	0.000
Catelliglobosispora	0.120	0.230	1.000	1.800	0.980	0.670	1.000	1.000	1.000	0.002	0.000	0.002	0.014	0.002	0.000	0.000	0.000	0.000	0.000	0.000
Aeromonas	0.220	0.400	1.000	1.500	1.000	1.000	1.000	0.940	0.280	0.002	0.000	0.000	0.000	0.000	0.001	0.002	0.000	0.003	0.000	0.012
Actinomyces	0.510	0.570	1.000	0.940	1.000	1.000	1.000	0.520	1.000	0.002	0.000	0.001	0.000	0.000	0.000	0.000	0.000	0.017	0.000	0.000
Isosphaera	0.018	0.047	1.000	3.000	1.000	1.000	0.120	1.000	1.000	0.002	0.000	0.000	0.000	0.000	0.017	0.001	0.000	0.000	0.000	0.000
Candidatus_Paracaedibacter	0.030	0.070	1.000	2.700	0.068	0.960	1.000	1.000	1.000	0.002	0.000	0.012	0.004	0.001	0.000	0.000	0.000	0.000	0.000	0.000
Aerococcus	0.001	0.003	0.280	5.600	1.000	1.000	1.000	1.000	0.540	0.002	0.000	0.000	0.006	0.009	0.000	0.000	0.000	0.000	0.000	0.002
Melghirimyces	0.470	0.560	1.000	1.000	0.470	1.000	1.000	1.000	1.000	0.002	0.017	0.000	0.000	0.000	0.000	0.000	0.000	0.000	0.000	0.000
Lachnoclostridium	0.470	0.560	1.000	1.000	1.000	1.000	1.000	0.470	1.000	0.002	0.000	0.000	0.000	0.000	0.000	0.000	0.000	0.017	0.000	0.000
Hydrogenophaga	0.470	0.560	1.000	1.000	1.000	0.470	1.000	1.000	1.000	0.002	0.000	0.000	0.017	0.000	0.000	0.000	0.000	0.000	0.000	0.000
Clostridiisalibacter	0.470	0.560	1.000	1.000	1.000	1.000	1.000	0.470	1.000	0.002	0.000	0.000	0.000	0.000	0.000	0.000	0.017	0.000	0.000	0.000
possible_genus_04	0.000	0.000	0.032	7.800	0.920	0.015	1.000	1.000	1.000	0.002	0.000	0.001	0.014	0.002	0.000	0.000	0.000	0.000	0.000	0.000
uncultured_Roseobacter_sp.	0.001	0.006	0.480	5.100	1.000	1.000	1.000	1.000	1.000	0.002	0.000	0.000	0.008	0.008	0.000	0.000	0.000	0.000	0.000	0.000
Telmatocola	0.002	0.010	0.930	4.600	1.000	1.000	1.000	1.000	1.000	0.002	0.000	0.000	0.007	0.009	0.000	0.000	0.000	0.000	0.000	0.000
uncultured_Caldilineaceae_bacterium	0.030	0.070	1.000	2.700	0.033	1.000	1.000	1.000	1.000	0.001	0.000	0.014	0.000	0.000	0.000	0.000	0.000	0.000	0.000	0.000
Lachnotalea	0.540	0.570	1.000	0.900	1.000	0.670	0.970	1.000	1.000	0.001	0.000	0.000	0.010	0.000	0.004	0.000	0.000	0.000	0.000	0.000
Chryseolinea	0.160	0.300	1.000	1.700	0.780	0.620	1.000	1.000	1.000	0.001	0.000	0.004	0.009	0.001	0.000	0.000	0.000	0.000	0.000	0.000

	One-way ANOVA				P values of Tukey’s HSD post hoc					Average relative abundance among plant compartments and fertilizer treatments (C: control, N: fertilized)										
Taxa	P Group	FDR Group	P adjusted Group	F Value Group	BS	Rh	R	Stem	Leaf	Average	BS-C mean	BS-N mean	Rh-C mean	Rh-N mean	R-C mean	R-N mean	Stem-C mean	Stem-N mean	Leaf-C mean	Leaf-N mean
Phaselicystis	0.007	0.025	1.000	3.700	0.013	0.850	1.000	1.000	1.000	0.001	0.000	0.012	0.002	0.000	0.000	0.000	0.000	0.000	0.000	0.000
uncultured_gamma_proteobacterium	0.230	0.410	1.000	1.500	0.810	1.000	1.000	1.000	1.000	0.001	0.000	0.004	0.003	0.006	0.000	0.000	0.000	0.000	0.000	0.000
uncultured_Solirubrobacter_sp.	0.002	0.008	0.640	4.900	0.980	0.990	1.000	1.000	1.000	0.001	0.000	0.001	0.005	0.008	0.000	0.000	0.000	0.000	0.000	0.000
Sandaracinus	0.035	0.081	1.000	2.600	0.980	0.044	1.000	1.000	1.000	0.001	0.000	0.001	0.012	0.000	0.000	0.000	0.000	0.000	0.000	0.000
Rubrivivax	0.046	0.100	1.000	2.400	0.095	0.750	1.000	1.000	1.000	0.001	0.000	0.010	0.003	0.000	0.000	0.000	0.000	0.000	0.000	0.000
Crossiella	0.027	0.064	1.000	2.800	0.037	0.980	1.000	1.000	1.000	0.001	0.000	0.012	0.001	0.000	0.000	0.000	0.000	0.000	0.000	0.000
uncultured_Thermomicrobia_bacterium	0.038	0.087	1.000	2.600	0.110	0.950	1.000	1.000	1.000	0.001	0.000	0.008	0.004	0.001	0.000	0.000	0.000	0.000	0.000	0.000
Bacteriovorax	0.000	0.000	0.000	21.000	1.000	0.190	1.000	1.000	1.000	0.001	0.000	0.000	0.009	0.004	0.000	0.000	0.000	0.000	0.000	0.000
Candidatus_Uhrbacteria_bacterium_RIFOXYC12_FULL_57_11	0.063	0.130	1.000	2.200	0.067	1.000	1.000	1.000	1.000	0.001	0.000	0.012	0.000	0.000	0.000	0.000	0.000	0.000	0.000	0.000
uncultured_Planctomycetaceae_bacterium	0.003	0.013	1.000	4.300	0.680	0.056	1.000	1.000	1.000	0.001	0.000	0.002	0.010	0.001	0.000	0.000	0.000	0.000	0.000	0.000
Siphonobacter	0.010	0.029	1.000	3.500	1.000	0.036	1.000	1.000	1.000	0.001	0.000	0.000	0.012	0.000	0.000	0.000	0.000	0.000	0.000	0.000
uncultured_candidate_division_SPAM_bacterium	0.005	0.018	1.000	4.000	0.007	1.000	1.000	1.000	1.000	0.001	0.000	0.012	0.000	0.000	0.000	0.000	0.000	0.000	0.000	0.000
G12WMSP1	0.010	0.029	1.000	3.400	0.014	1.000	1.000	1.000	1.000	0.001	0.000	0.012	0.000	0.000	0.000	0.000	0.000	0.000	0.000	0.000
bacterium_Ellin6529	0.400	0.560	1.000	1.100	0.590	0.990	1.000	1.000	1.000	0.001	0.000	0.008	0.004	0.001	0.000	0.000	0.000	0.000	0.000	0.000
Sporocytophaga	0.190	0.350	1.000	1.600	1.000	0.880	0.980	1.000	1.000	0.001	0.000	0.000	0.009	0.002	0.002	0.000	0.000	0.000	0.000	0.000
Shimazuella	0.670	0.670	1.000	0.740	1.000	1.000	1.000	0.770	1.000	0.001	0.000	0.000	0.000	0.000	0.003	0.001	0.000	0.006	0.001	0.001
UTCFX1	0.570	0.590	1.000	0.860	0.950	1.000	1.000	1.000	1.000	0.001	0.009	0.001	0.001	0.000	0.000	0.000	0.000	0.000	0.000	0.000
Hydrogenispora	0.600	0.610	1.000	0.830	0.910	1.000	1.000	0.800	1.000	0.001	0.000	0.004	0.001	0.000	0.000	0.000	0.006	0.000	0.000	0.000
Wolbachia	0.036	0.083	1.000	2.600	0.980	0.048	1.000	1.000	1.000	0.001	0.000	0.001	0.010	0.000	0.000	0.000	0.000	0.000	0.000	0.000
Roseateles	0.150	0.280	1.000	1.700	0.860	0.700	1.000	1.000	1.000	0.001	0.000	0.003	0.008	0.001	0.000	0.000	0.000	0.000	0.000	0.000
Dinghuibacter	0.000	0.000	0.000	22.000	1.000	0.000	1.000	1.000	1.000	0.001	0.000	0.000	0.010	0.000	0.000	0.000	0.000	0.000	0.000	0.000
uncultured_Holophagae_bacterium	0.470	0.560	1.000	1.000	0.470	1.000	1.000	1.000	1.000	0.001	0.000	0.010	0.000	0.000	0.000	0.000	0.000	0.000	0.000	0.000
uncultured_Thermosporothrix_sp.	0.010	0.029	1.000	3.400	0.027	0.570	1.000	1.000	1.000	0.001	0.000	0.008	0.002	0.000	0.000	0.000	0.000	0.000	0.000	0.000
Janthinobacterium	0.550	0.570	1.000	0.900	0.940	1.000	1.000	0.710	1.000	0.001	0.000	0.003	0.000	0.000	0.000	0.000	0.006	0.000	0.000	0.000
Micropepsis	0.560	0.580	1.000	0.870	0.570	1.000	1.000	1.000	1.000	0.001	0.000	0.008	0.001	0.001	0.000	0.000	0.000	0.000	0.000	0.000
X19591	0.390	0.560	1.000	1.100	0.630	0.990	1.000	1.000	1.000	0.001	0.000	0.005	0.004	0.001	0.000	0.000	0.000	0.000	0.000	0.000
Edaphochlorella_mirabilis	0.006	0.020	1.000	3.900	1.000	0.008	1.000	1.000	1.000	0.001	0.000	0.000	0.009	0.000	0.000	0.000	0.000	0.000	0.000	0.000

	One-way ANOVA				P values of Tukey's HSD post hoc					Average relative abundance among plant compartments and fertilizer treatments (C: control, N: fertilized)										
Taxa	P Group	FDR Group	P adjusted Group	F Value Group	BS	Rh	R	Stem	Leaf	Average	BS-C mean	BS-N mean	Rh-C mean	Rh-N mean	R-C mean	R-N mean	Stem-C mean	Stem-N mean	Leaf-C mean	Leaf-N mean
Maribius	0.470	0.560	1.000	1.000	1.000	1.000	1.000	0.470	1.000	0.001	0.000	0.000	0.000	0.000	0.000	0.000	0.009	0.000	0.000	0.000
Kocuria	0.470	0.560	1.000	1.000	0.470	1.000	1.000	1.000	1.000	0.001	0.009	0.000	0.000	0.000	0.000	0.000	0.000	0.000	0.000	0.000
FFCH7168	0.015	0.041	1.000	3.200	1.000	1.000	1.000	1.000	1.000	0.001	0.000	0.000	0.006	0.003	0.000	0.000	0.000	0.000	0.000	0.000
C39	0.510	0.570	1.000	0.950	1.000	1.000	1.000	0.510	1.000	0.001	0.000	0.000	0.001	0.000	0.000	0.000	0.000	0.009	0.000	0.000
Pelosinus	0.550	0.570	1.000	0.890	1.000	0.730	0.930	1.000	1.000	0.001	0.000	0.000	0.006	0.000	0.003	0.000	0.000	0.000	0.000	0.000
uncultured_Desulfovirga_sp.	0.011	0.032	1.000	3.400	0.015	1.000	1.000	1.000	1.000	0.001	0.000	0.009	0.000	0.000	0.000	0.000	0.000	0.000	0.000	0.000
Oceanobacillus	0.670	0.670	1.000	0.730	0.990	1.000	1.000	0.760	1.000	0.001	0.000	0.002	0.001	0.001	0.000	0.000	0.005	0.000	0.000	0.000
Ferruginibacter	0.014	0.039	1.000	3.200	0.017	1.000	1.000	1.000	1.000	0.001	0.000	0.009	0.000	0.000	0.000	0.000	0.000	0.000	0.000	0.000
Segetibacter	0.013	0.037	1.000	3.300	1.000	0.980	1.000	1.000	1.000	0.001	0.000	0.000	0.006	0.003	0.000	0.000	0.000	0.000	0.000	0.000
bacterium_enrichment_culture_clone_auto10_4W	0.510	0.570	1.000	0.950	0.510	1.000	1.000	1.000	1.000	0.001	0.000	0.008	0.001	0.000	0.000	0.000	0.000	0.000	0.000	0.000
Crenobacter	0.190	0.350	1.000	1.600	0.900	0.800	1.000	1.000	1.000	0.001	0.000	0.002	0.006	0.001	0.000	0.000	0.000	0.000	0.000	0.000
uncultured_Archangiaceae_bacterium	0.014	0.039	1.000	3.200	0.670	0.190	1.000	1.000	1.000	0.001	0.000	0.002	0.006	0.000	0.000	0.000	0.000	0.000	0.000	0.000
Anaeromyxobacter_dehalogenans	0.044	0.098	1.000	2.500	0.058	0.980	1.000	1.000	1.000	0.001	0.000	0.008	0.001	0.000	0.000	0.000	0.000	0.000	0.000	0.000
Candidatus_Methylomirabilis	0.022	0.055	1.000	2.900	0.043	0.980	1.000	1.000	1.000	0.001	0.000	0.006	0.002	0.000	0.000	0.000	0.000	0.000	0.000	0.000
Anaerospora	0.530	0.570	1.000	0.920	1.000	0.580	0.990	1.000	1.000	0.001	0.000	0.000	0.007	0.000	0.001	0.000	0.000	0.000	0.000	0.000
Planctomycetales_bacterium_Ellin7224	0.083	0.160	1.000	2.100	0.830	0.150	1.000	1.000	1.000	0.001	0.000	0.002	0.006	0.000	0.000	0.000	0.000	0.000	0.000	0.000
Microgenomates_group_bacterium_RIFCSPLOWO2_01_FULL_47_10	0.091	0.180	1.000	2.000	1.000	0.340	1.000	1.000	1.000	0.001	0.000	0.000	0.008	0.000	0.000	0.000	0.000	0.000	0.000	0.000
uncultured_Bellilinea_sp.	0.540	0.570	1.000	0.900	1.000	1.000	1.000	1.000	1.000	0.001	0.006	0.002	0.000	0.000	0.000	0.000	0.000	0.000	0.000	0.000
Kaistia	0.610	0.620	1.000	0.820	1.000	0.990	0.990	1.000	1.000	0.001	0.000	0.000	0.002	0.000	0.001	0.005	0.000	0.000	0.000	0.000
Parcubacteria_bacterium_C7867005	0.009	0.028	1.000	3.500	1.000	0.630	1.000	1.000	1.000	0.001	0.000	0.000	0.006	0.002	0.000	0.000	0.000	0.000	0.000	0.000
uncultured_Verrucomicrobia_subdivision_3_bacterium	0.062	0.130	1.000	2.300	1.000	0.850	1.000	1.000	1.000	0.001	0.000	0.000	0.006	0.002	0.000	0.000	0.000	0.000	0.000	0.000
Litorilinea	0.320	0.560	1.000	1.300	0.610	1.000	1.000	1.000	1.000	0.001	0.000	0.004	0.003	0.001	0.000	0.000	0.000	0.000	0.000	0.000
Nitrosomonas	0.068	0.140	1.000	2.200	0.170	0.620	1.000	1.000	1.000	0.001	0.000	0.005	0.003	0.000	0.000	0.000	0.000	0.000	0.000	0.000
Candidatus_Kaiserbacteria_bacterium_RIFOXYB1_FULL_46_14	0.022	0.055	1.000	2.900	1.000	0.300	1.000	1.000	1.000	0.001	0.000	0.000	0.007	0.001	0.000	0.000	0.000	0.000	0.000	0.000
Syntrophobacter	0.470	0.560	1.000	1.000	0.470	1.000	1.000	1.000	1.000	0.001	0.008	0.000	0.000	0.000	0.000	0.000	0.000	0.000	0.000	0.000

	One-way ANOVA				P values of Tukey’s HSD post hoc					Average relative abundance among plant compartments and fertilizer treatments (C: control, N: fertilized)										
Taxa	P Group	FDR Group	P adjusted Group	F Value Group	BS	Rh	R	Stem	Leaf	Average	BS-C mean	BS-N mean	Rh-C mean	Rh-N mean	R-C mean	R-N mean	Stem-C mean	Stem-N mean	Leaf-C mean	Leaf-N mean
Serratia	0.470	0.560	1.000	1.000	0.470	1.000	1.000	1.000	1.000	0.001	0.000	0.008	0.000	0.000	0.000	0.000	0.000	0.000	0.000	0.000
CENA518	0.000	0.003	0.200	6.000	1.000	0.078	1.000	1.000	1.000	0.001	0.000	0.000	0.006	0.001	0.000	0.000	0.000	0.000	0.000	0.000
uncultured_Planctomycetales_bacterium	0.510	0.570	1.000	0.950	0.500	1.000	1.000	1.000	1.000	0.001	0.000	0.007	0.000	0.000	0.000	0.000	0.000	0.000	0.000	0.000
bacterium_Ellin6515	0.005	0.018	1.000	4.000	0.007	1.000	1.000	1.000	1.000	0.001	0.000	0.007	0.000	0.000	0.000	0.000	0.000	0.000	0.000	0.000
Ammoniibacillus	0.540	0.570	1.000	0.900	0.520	1.000	1.000	1.000	1.000	0.001	0.000	0.006	0.000	0.000	0.000	0.000	0.000	0.000	0.000	0.000
Methylibium	0.005	0.019	1.000	3.900	1.000	0.008	1.000	1.000	1.000	0.001	0.000	0.000	0.006	0.000	0.000	0.000	0.000	0.000	0.000	0.000
Oligoflexus	0.470	0.560	1.000	1.000	1.000	0.470	1.000	1.000	1.000	0.001	0.000	0.000	0.006	0.000	0.000	0.000	0.000	0.000	0.000	0.000
uncultured_Iamia_sp.	0.009	0.027	1.000	3.600	1.000	0.990	1.000	1.000	1.000	0.001	0.000	0.000	0.004	0.002	0.000	0.000	0.000	0.000	0.000	0.000
Aridibacter	0.066	0.140	1.000	2.200	1.000	0.610	1.000	1.000	1.000	0.001	0.000	0.000	0.005	0.001	0.000	0.000	0.000	0.000	0.000	0.000
Ruminiclostridium_1	0.260	0.460	1.000	1.400	1.000	0.280	1.000	1.000	1.000	0.001	0.000	0.000	0.005	0.000	0.001	0.000	0.000	0.000	0.000	0.000
Caedibacter	0.650	0.650	1.000	0.760	0.770	1.000	1.000	1.000	1.000	0.001	0.000	0.003	0.000	0.000	0.002	0.000	0.000	0.000	0.000	0.000
Caenimonas	0.047	0.100	1.000	2.400	1.000	0.710	1.000	1.000	1.000	0.001	0.000	0.000	0.004	0.001	0.000	0.000	0.000	0.000	0.000	0.000
Marmoricola	0.008	0.025	1.000	3.600	1.000	0.011	1.000	1.000	1.000	0.001	0.000	0.000	0.005	0.000	0.000	0.000	0.000	0.000	0.000	0.000
uncultured_Rubrobacterales_bacterium	0.470	0.560	1.000	1.000	0.470	1.000	1.000	1.000	1.000	0.001	0.000	0.005	0.000	0.000	0.000	0.000	0.000	0.000	0.000	0.000
Cloacibacterium	0.470	0.560	1.000	1.000	1.000	1.000	1.000	1.000	0.470	0.001	0.000	0.000	0.000	0.000	0.000	0.000	0.000	0.000	0.005	0.000
Candidatus_Kapabacteria_sp._5999	0.470	0.560	1.000	1.000	1.000	0.470	1.000	1.000	1.000	0.001	0.000	0.000	0.005	0.000	0.000	0.000	0.000	0.000	0.000	0.000
Candidatus_Saccharimonas	0.550	0.570	1.000	0.890	1.000	0.790	0.890	1.000	1.000	0.001	0.000	0.000	0.003	0.000	0.002	0.000	0.000	0.000	0.000	0.000
Comamonas	0.005	0.018	1.000	4.000	1.000	0.007	1.000	1.000	1.000	0.000	0.000	0.000	0.000	0.005	0.000	0.000	0.000	0.000	0.000	0.000
uncultured_Micromonospora_sp.	0.470	0.560	1.000	1.000	0.470	1.000	1.000	1.000	1.000	0.000	0.000	0.005	0.000	0.000	0.000	0.000	0.000	0.000	0.000	0.000
uncultured_Bacteroidetes_bacterium	0.000	0.000	0.020	8.400	1.000	0.007	1.000	1.000	1.000	0.000	0.000	0.000	0.004	0.000	0.000	0.000	0.000	0.000	0.000	0.000
Thermoactinomyces	0.550	0.570	1.000	0.890	1.000	1.000	1.000	0.820	0.870	0.000	0.000	0.000	0.000	0.000	0.000	0.000	0.003	0.000	0.000	0.002
Acidobacteria_bacterium_IGE010	0.610	0.620	1.000	0.810	1.000	0.990	0.970	1.000	1.000	0.000	0.000	0.000	0.003	0.001	0.001	0.000	0.000	0.000	0.000	0.000
uncultured_Bacteriovorax_sp.	0.077	0.160	1.000	2.100	1.000	0.800	0.140	1.000	1.000	0.000	0.000	0.000	0.001	0.000	0.004	0.000	0.000	0.000	0.000	0.000
Parapedobacter	0.500	0.570	1.000	0.960	1.000	0.490	1.000	1.000	1.000	0.000	0.000	0.000	0.004	0.000	0.000	0.000	0.000	0.000	0.000	0.000
uncultured_bacterium_GRWP3330	0.470	0.560	1.000	1.000	0.880	1.000	1.000	1.000	1.000	0.000	0.000	0.002	0.002	0.001	0.000	0.000	0.000	0.000	0.000	0.000
uncultured_Gaiella_sp.	0.000	0.000	0.000	31.000	1.000	0.000	1.000	1.000	1.000	0.000	0.000	0.000	0.004	0.000	0.000	0.000	0.000	0.000	0.000	0.000
Tahibacter	0.470	0.560	1.000	1.000	1.000	0.470	1.000	1.000	1.000	0.000	0.000	0.000	0.004	0.000	0.000	0.000	0.000	0.000	0.000	0.000
uncultured_archaeon	0.038	0.087	1.000	2.600	0.048	1.000	1.000	1.000	1.000	0.000	0.000	0.004	0.000	0.000	0.000	0.000	0.000	0.000	0.000	0.000

	One-way ANOVA				P values of Tukey's HSD post hoc					Average relative abundance among plant compartments and fertilizer treatments (C: control, N: fertilized)										
Taxa	P Group	FDR Group	P adjusted Group	F Value Group	BS	Rh	R	Stem	Leaf	Average	BS-C mean	BS-N mean	Rh-C mean	Rh-N mean	R-C mean	R-N mean	Stem-C mean	Stem-N mean	Leaf-C mean	Leaf-N mean
Hyaloperonospora_arabidopsidis	0.180	0.330	1.000	1.600	1.000	1.000	1.000	1.000	1.000	0.000	0.000	0.000	0.002	0.003	0.000	0.000	0.000	0.000	0.000	0.000
Polyangium	0.520	0.570	1.000	0.940	0.530	1.000	1.000	1.000	1.000	0.000	0.000	0.004	0.000	0.000	0.000	0.000	0.000	0.000	0.000	0.000
bacterium_Ellin6537	0.530	0.570	1.000	0.920	0.580	0.990	1.000	1.000	1.000	0.000	0.000	0.004	0.000	0.001	0.000	0.000	0.000	0.000	0.000	0.000
Brevifollis	0.510	0.570	1.000	0.950	1.000	1.000	0.510	1.000	1.000	0.000	0.000	0.000	0.000	0.000	0.004	0.000	0.000	0.000	0.000	0.000
uncultured_soil_bacterium_PBS22	0.150	0.280	1.000	1.700	0.780	1.000	1.000	1.000	1.000	0.000	0.000	0.001	0.002	0.001	0.000	0.000	0.000	0.000	0.000	0.000
Candidatus_Cardinium	0.690	0.690	1.000	0.710	0.970	1.000	1.000	1.000	1.000	0.000	0.000	0.001	0.001	0.002	0.000	0.000	0.000	0.000	0.000	0.000
uncultured_Thermoplasmatales_archaeon	0.590	0.610	1.000	0.840	0.650	1.000	1.000	1.000	1.000	0.000	0.000	0.003	0.001	0.000	0.000	0.000	0.000	0.000	0.000	0.000
Candidatus_Amoebophilus	0.500	0.570	1.000	0.950	0.500	1.000	1.000	1.000	1.000	0.000	0.000	0.004	0.000	0.000	0.000	0.000	0.000	0.000	0.000	0.000
Ga0074140	0.470	0.560	1.000	1.000	1.000	0.470	1.000	1.000	1.000	0.000	0.000	0.000	0.004	0.000	0.000	0.000	0.000	0.000	0.000	0.000
Cytophaga	0.470	0.560	1.000	1.000	1.000	0.470	1.000	1.000	1.000	0.000	0.000	0.000	0.004	0.000	0.000	0.000	0.000	0.000	0.000	0.000
CyanobacteriaMelainabacteria_group_bacterium_S15BMN24_CBMW_12	0.012	0.034	1.000	3.300	1.000	0.970	1.000	1.000	1.000	0.000	0.000	0.000	0.001	0.003	0.000	0.000	0.000	0.000	0.000	0.000
Ferrovibrio	0.600	0.610	1.000	0.820	1.000	0.980	0.950	1.000	1.000	0.000	0.000	0.000	0.002	0.000	0.001	0.000	0.000	0.000	0.000	0.000
Peptoniphilus	0.550	0.570	1.000	0.890	0.920	1.000	1.000	1.000	0.750	0.000	0.000	0.001	0.000	0.000	0.000	0.000	0.000	0.000	0.000	0.002
Fluviicola	0.510	0.570	1.000	0.940	1.000	0.870	1.000	1.000	1.000	0.000	0.000	0.000	0.003	0.000	0.000	0.000	0.000	0.000	0.000	0.000
Deinococcus	0.500	0.570	1.000	0.960	1.000	1.000	1.000	1.000	0.490	0.000	0.000	0.000	0.000	0.000	0.000	0.000	0.000	0.000	0.000	0.003
Terriglobus	0.550	0.570	1.000	0.890	1.000	0.940	0.720	1.000	1.000	0.000	0.000	0.000	0.000	0.001	0.000	0.002	0.000	0.000	0.000	0.000
Panacagrimonas	0.005	0.018	1.000	4.000	1.000	0.007	1.000	1.000	1.000	0.000	0.000	0.000	0.003	0.000	0.000	0.000	0.000	0.000	0.000	0.000
Thermomonas	0.470	0.560	1.000	1.000	1.000	0.470	1.000	1.000	1.000	0.000	0.000	0.000	0.003	0.000	0.000	0.000	0.000	0.000	0.000	0.000
Rhodobacter	0.470	0.560	1.000	1.000	1.000	0.470	1.000	1.000	1.000	0.000	0.000	0.000	0.003	0.000	0.000	0.000	0.000	0.000	0.000	0.000
Luteitalea	0.470	0.560	1.000	1.000	1.000	0.470	1.000	1.000	1.000	0.000	0.000	0.000	0.003	0.000	0.000	0.000	0.000	0.000	0.000	0.000
Anaerovorax	0.520	0.570	1.000	0.930	1.000	0.550	1.000	1.000	1.000	0.000	0.000	0.000	0.003	0.000	0.000	0.000	0.000	0.000	0.000	0.000
Salinicola	0.540	0.570	1.000	0.900	1.000	0.960	0.680	1.000	1.000	0.000	0.000	0.000	0.001	0.000	0.002	0.000	0.000	0.000	0.000	0.000
uncultured_Candidatus_Saccharibacteria_bacterium	0.550	0.570	1.000	0.890	0.740	0.920	1.000	1.000	1.000	0.000	0.000	0.002	0.001	0.000	0.000	0.000	0.000	0.000	0.000	0.000
Haemophilus	0.470	0.560	1.000	1.000	0.470	1.000	1.000	1.000	1.000	0.000	0.000	0.003	0.000	0.000	0.000	0.000	0.000	0.000	0.000	0.000
Gaiella_sp._EBR4RS1	0.470	0.560	1.000	1.000	0.470	1.000	1.000	1.000	1.000	0.000	0.000	0.003	0.000	0.000	0.000	0.000	0.000	0.000	0.000	0.000
JTB255_marine_benthic_group	0.520	0.570	1.000	0.930	0.530	1.000	1.000	1.000	1.000	0.000	0.000	0.003	0.000	0.000	0.000	0.000	0.000	0.000	0.000	0.000
Kroppenstedtia	0.510	0.570	1.000	0.940	0.510	1.000	1.000	1.000	1.000	0.000	0.000	0.003	0.000	0.000	0.000	0.000	0.000	0.000	0.000	0.000
Candidatus_Renichlamydia	0.610	0.620	1.000	0.820	0.900	0.830	1.000	1.000	1.000	0.000	0.000	0.001	0.001	0.000	0.000	0.000	0.000	0.000	0.000	0.000
bacterium_Ellin517	0.017	0.045	1.000	3.100	1.000	0.110	1.000	1.000	1.000	0.000	0.000	0.000	0.003	0.000	0.000	0.000	0.000	0.000	0.000	0.000

	One-way ANOVA				P values of Tukey's HSD post hoc					Average relative abundance among plant compartments and fertilizer treatments (C: control, N: fertilized)										
Taxa	P Group	FDR Group	P adjusted Group	F Value Group	BS	Rh	R	Stem	Leaf	Average	BS-C mean	BS-N mean	Rh-C mean	Rh-N mean	R-C mean	R-N mean	Stem-C mean	Stem-N mean	Leaf-C mean	Leaf-N mean
uncultured_Rubrobacteraceae_bacterium	0.540	0.570	1.000	0.900	0.670	0.970	1.000	1.000	1.000	0.000	0.000	0.002	0.001	0.000	0.000	0.000	0.000	0.000	0.000	0.000
Fimbriimonas	0.540	0.570	1.000	0.900	1.000	0.670	0.970	1.000	1.000	0.000	0.000	0.000	0.002	0.000	0.001	0.000	0.000	0.000	0.000	0.000
uncultured_bacterium_KFJG30B11	0.005	0.019	1.000	3.900	1.000	0.007	1.000	1.000	1.000	0.000	0.000	0.000	0.003	0.000	0.000	0.000	0.000	0.000	0.000	0.000
uncultured_bacterium_mle125	0.009	0.027	1.000	3.600	1.000	0.012	1.000	1.000	1.000	0.000	0.000	0.000	0.003	0.000	0.000	0.000	0.000	0.000	0.000	0.000
uncultured_Desulfuromonadales_bacterium	0.470	0.560	1.000	1.000	0.470	1.000	1.000	1.000	1.000	0.000	0.000	0.003	0.000	0.000	0.000	0.000	0.000	0.000	0.000	0.000
Nubsella	0.470	0.560	1.000	1.000	1.000	0.470	1.000	1.000	1.000	0.000	0.000	0.000	0.003	0.000	0.000	0.000	0.000	0.000	0.000	0.000
Emticicia	0.470	0.560	1.000	1.000	1.000	0.470	1.000	1.000	1.000	0.000	0.000	0.000	0.003	0.000	0.000	0.000	0.000	0.000	0.000	0.000
Candidatus_Adlerbacteria_bacterium_GW2011_GWC1_50_9	0.470	0.560	1.000	1.000	0.470	1.000	1.000	1.000	1.000	0.000	0.000	0.003	0.000	0.000	0.000	0.000	0.000	0.000	0.000	0.000
Nannocystis	0.550	0.570	1.000	0.890	1.000	0.730	0.930	1.000	1.000	0.000	0.000	0.000	0.002	0.000	0.001	0.000	0.000	0.000	0.000	0.000
Solimonas	0.120	0.230	1.000	1.900	1.000	0.990	1.000	1.000	1.000	0.000	0.000	0.000	0.002	0.001	0.000	0.000	0.000	0.000	0.000	0.000
Candidatus_Omnitrophus	0.580	0.600	1.000	0.860	0.600	1.000	1.000	1.000	1.000	0.000	0.000	0.002	0.000	0.000	0.000	0.000	0.000	0.000	0.000	0.000
Actinospica	0.550	0.570	1.000	0.890	0.910	0.770	1.000	1.000	1.000	0.000	0.000	0.001	0.001	0.000	0.000	0.000	0.000	0.000	0.000	0.000
uncultured_Sphaerobacter_sp.	0.005	0.018	1.000	4.000	1.000	0.007	1.000	1.000	1.000	0.000	0.000	0.000	0.002	0.000	0.000	0.000	0.000	0.000	0.000	0.000
Roseiflexus	0.005	0.018	1.000	4.000	1.000	0.007	1.000	1.000	1.000	0.000	0.000	0.000	0.002	0.000	0.000	0.000	0.000	0.000	0.000	0.000
Tepidisphaera	0.014	0.039	1.000	3.200	1.000	0.019	1.000	1.000	1.000	0.000	0.000	0.000	0.002	0.000	0.000	0.000	0.000	0.000	0.000	0.000
Sorangium	0.470	0.560	1.000	1.000	0.470	1.000	1.000	1.000	1.000	0.000	0.000	0.002	0.000	0.000	0.000	0.000	0.000	0.000	0.000	0.000
hgcI_clade	0.470	0.560	1.000	1.000	1.000	1.000	1.000	1.000	0.470	0.000	0.000	0.000	0.000	0.000	0.000	0.000	0.000	0.000	0.000	0.002
Candidatus_Profftella	0.470	0.560	1.000	1.000	1.000	0.470	1.000	1.000	1.000	0.000	0.000	0.000	0.002	0.000	0.000	0.000	0.000	0.000	0.000	0.000
Candidatus_Alysiosphaera	0.470	0.560	1.000	1.000	0.470	1.000	1.000	1.000	1.000	0.000	0.000	0.002	0.000	0.000	0.000	0.000	0.000	0.000	0.000	0.000
Hirschia	0.550	0.570	1.000	0.890	0.840	0.840	1.000	1.000	1.000	0.000	0.000	0.001	0.001	0.000	0.000	0.000	0.000	0.000	0.000	0.000
Pontibacter	0.070	0.140	1.000	2.200	1.000	0.860	1.000	1.000	1.000	0.000	0.000	0.000	0.002	0.000	0.000	0.000	0.000	0.000	0.000	0.000
uncultured_Crater_Lake_bacterium_CL50015	0.380	0.560	1.000	1.100	1.000	1.000	1.000	1.000	1.000	0.000	0.000	0.000	0.001	0.001	0.000	0.000	0.000	0.000	0.000	0.000
Phycisphaera	0.005	0.019	1.000	3.900	1.000	0.008	1.000	1.000	1.000	0.000	0.000	0.000	0.002	0.000	0.000	0.000	0.000	0.000	0.000	0.000
Chitinimonas	0.005	0.019	1.000	3.900	1.000	0.008	1.000	1.000	1.000	0.000	0.000	0.000	0.002	0.000	0.000	0.000	0.000	0.000	0.000	0.000
Persicitalea	0.470	0.560	1.000	1.000	1.000	0.470	1.000	1.000	1.000	0.000	0.000	0.000	0.002	0.000	0.000	0.000	0.000	0.000	0.000	0.000
Exiguobacterium	0.077	0.160	1.000	2.100	1.000	0.920	1.000	1.000	1.000	0.000	0.000	0.000	0.001	0.000	0.000	0.000	0.000	0.000	0.000	0.000
uncultured_Sphingobacteriales_bacterium	0.550	0.570	1.000	0.890	1.000	1.000	1.000	1.000	1.000	0.000	0.000	0.000	0.001	0.001	0.000	0.000	0.000	0.000	0.000	0.000

	One-way ANOVA				P values of Tukey's HSD post hoc					Average relative abundance among plant compartments and fertilizer treatments (C: control, N: fertilized)										
Taxa	P Group	FDR Group	P adjusted Group	F Value Group	BS	Rh	R	Stem	Leaf	Average	BS-C mean	BS-N mean	Rh-C mean	Rh-N mean	R-C mean	R-N mean	Stem-C mean	Stem-N mean	Leaf-C mean	Leaf-N mean
bacterium_enrichment_culture_clone_B302011	0.550	0.570	1.000	0.900	0.710	0.950	1.000	1.000	1.000	0.000	0.000	0.001	0.001	0.000	0.000	0.000	0.000	0.000	0.000	0.000
Azohydromonas	0.550	0.570	1.000	0.900	1.000	1.000	1.000	1.000	1.000	0.000	0.000	0.000	0.001	0.001	0.000	0.000	0.000	0.000	0.000	0.000
uncultured_Halanaerobiaceae_bacterium	0.005	0.018	1.000	4.000	1.000	0.007	1.000	1.000	1.000	0.000	0.000	0.000	0.000	0.002	0.000	0.000	0.000	0.000	0.000	0.000
Virgisporangium	0.470	0.560	1.000	1.000	1.000	0.470	1.000	1.000	1.000	0.000	0.000	0.000	0.002	0.000	0.000	0.000	0.000	0.000	0.000	0.000
Jiangella	0.470	0.560	1.000	1.000	1.000	0.470	1.000	1.000	1.000	0.000	0.000	0.000	0.000	0.002	0.000	0.000	0.000	0.000	0.000	0.000
Herbaspirillum	0.470	0.560	1.000	1.000	0.470	1.000	1.000	1.000	1.000	0.000	0.000	0.002	0.000	0.000	0.000	0.000	0.000	0.000	0.000	0.000
Caldalkalibacillus	0.470	0.560	1.000	1.000	0.470	1.000	1.000	1.000	1.000	0.000	0.000	0.002	0.000	0.000	0.000	0.000	0.000	0.000	0.000	0.000
uncultured_Pedosphaera_sp.	0.550	0.570	1.000	0.890	1.000	1.000	1.000	1.000	1.000	0.000	0.000	0.000	0.001	0.001	0.000	0.000	0.000	0.000	0.000	0.000
Rickettsia	0.550	0.570	1.000	0.890	1.000	1.000	1.000	1.000	1.000	0.000	0.000	0.000	0.000	0.000	0.001	0.001	0.000	0.000	0.000	0.000
uncultured_Streptomyces_sp.	0.470	0.560	1.000	1.000	0.470	1.000	1.000	1.000	1.000	0.000	0.000	0.001	0.000	0.000	0.000	0.000	0.000	0.000	0.000	0.000
Sphingomonas	0.470	0.560	1.000	1.000	1.000	1.000	0.470	1.000	1.000	0.000	0.000	0.000	0.000	0.000	0.000	0.001	0.000	0.000	0.000	0.000
Roseiarcus	0.470	0.560	1.000	1.000	0.470	1.000	1.000	1.000	1.000	0.000	0.000	0.001	0.000	0.000	0.000	0.000	0.000	0.000	0.000	0.000
OLB12	0.470	0.560	1.000	1.000	0.470	1.000	1.000	1.000	1.000	0.000	0.000	0.001	0.000	0.000	0.000	0.000	0.000	0.000	0.000	0.000
Clostridium_sensu_stricto_18	0.470	0.560	1.000	1.000	1.000	1.000	0.470	1.000	1.000	0.000	0.000	0.000	0.000	0.000	0.001	0.000	0.000	0.000	0.000	0.000
Hymenobacter	0.120	0.230	1.000	1.800	1.000	1.000	1.000	1.000	1.000	0.000	0.000	0.000	0.000	0.001	0.000	0.000	0.000	0.000	0.000	0.000
Flaviaesturariibacter	0.550	0.570	1.000	0.890	1.000	1.000	1.000	1.000	1.000	0.000	0.000	0.000	0.001	0.001	0.000	0.000	0.000	0.000	0.000	0.000
Pigmentiphaga	0.470	0.560	1.000	1.000	1.000	1.000	0.470	1.000	1.000	0.000	0.000	0.000	0.000	0.000	0.001	0.000	0.000	0.000	0.000	0.000
OM27_clade	0.470	0.560	1.000	1.000	0.470	1.000	1.000	1.000	1.000	0.000	0.000	0.001	0.000	0.000	0.000	0.000	0.000	0.000	0.000	0.000
Myxococcales_bacterium_Gsoil_473	0.470	0.560	1.000	1.000	1.000	0.470	1.000	1.000	1.000	0.000	0.000	0.000	0.001	0.000	0.000	0.000	0.000	0.000	0.000	0.000
Methyloversatilis	0.470	0.560	1.000	1.000	1.000	0.470	1.000	1.000	1.000	0.000	0.000	0.000	0.001	0.000	0.000	0.000	0.000	0.000	0.000	0.000
groundwater_metagenome	0.470	0.560	1.000	1.000	1.000	0.470	1.000	1.000	1.000	0.000	0.000	0.000	0.001	0.000	0.000	0.000	0.000	0.000	0.000	0.000
Geobacter	0.470	0.560	1.000	1.000	0.470	1.000	1.000	1.000	1.000	0.000	0.000	0.001	0.000	0.000	0.000	0.000	0.000	0.000	0.000	0.000
Clostridium_sensu_stricto_11	0.470	0.560	1.000	1.000	1.000	1.000	0.470	1.000	1.000	0.000	0.000	0.000	0.000	0.000	0.001	0.000	0.000	0.000	0.000	0.000
Actinobacteria_bacterium_RBG_13_63_9	0.470	0.560	1.000	1.000	0.470	1.000	1.000	1.000	1.000	0.000	0.000	0.001	0.000	0.000	0.000	0.000	0.000	0.000	0.000	0.000
Acidisphaera	0.470	0.560	1.000	1.000	1.000	0.470	1.000	1.000	1.000	0.000	0.000	0.000	0.000	0.001	0.000	0.000	0.000	0.000	0.000	0.000
marine_metagenome	0.049	0.110	1.000	2.400	1.000	0.710	1.000	1.000	1.000	0.000	0.000	0.000	0.001	0.000	0.000	0.000	0.000	0.000	0.000	0.000
Segniliparus	0.550	0.570	1.000	0.890	1.000	0.780	0.890	1.000	1.000	0.000	0.000	0.000	0.001	0.000	0.000	0.000	0.000	0.000	0.000	0.000
Azoarcus	0.550	0.570	1.000	0.890	1.000	1.000	1.000	1.000	1.000	0.000	0.000	0.000	0.000	0.001	0.000	0.000	0.000	0.000	0.000	0.000
Desulfotomaculum	0.610	0.620	1.000	0.810	1.000	1.000	1.000	1.000	1.000	0.000	0.000	0.000	0.000	0.000	0.001	0.000	0.000	0.000	0.000	0.000

	One-way ANOVA				P values of Tukey’s HSD post hoc					Average relative abundance among plant compartments and fertilizer treatments (C: control, N: fertilized)										
Taxa	P Group	FDR Group	P adjusted Group	F Value Group	BS	Rh	R	Stem	Leaf	Average	BS-C mean	BS-N mean	Rh-C mean	Rh-N mean	R-C mean	R-N mean	Stem-C mean	Stem-N mean	Leaf-C mean	Leaf-N mean
Polaribacter	0.470	0.560	1.000	1.000	1.000	1.000	1.000	0.470	1.000	0.000	0.000	0.000	0.000	0.000	0.000	0.000	0.000	0.001	0.000	0.000
Paludisphaera	0.470	0.560	1.000	1.000	1.000	0.470	1.000	1.000	1.000	0.000	0.000	0.000	0.001	0.000	0.000	0.000	0.000	0.000	0.000	0.000
Micromonospora	0.470	0.560	1.000	1.000	0.470	1.000	1.000	1.000	1.000	0.000	0.000	0.001	0.000	0.000	0.000	0.000	0.000	0.000	0.000	0.000
Lutispora	0.470	0.560	1.000	1.000	1.000	0.470	1.000	1.000	1.000	0.000	0.000	0.000	0.001	0.000	0.000	0.000	0.000	0.000	0.000	0.000
bacterium_WX65	0.470	0.560	1.000	1.000	0.470	1.000	1.000	1.000	1.000	0.000	0.000	0.001	0.000	0.000	0.000	0.000	0.000	0.000	0.000	0.000
Peredibacter	0.630	0.640	1.000	0.790	1.000	1.000	0.990	1.000	1.000	0.000	0.000	0.000	0.000	0.000	0.000	0.000	0.000	0.000	0.000	0.000
Vulgatibacter	0.550	0.570	1.000	0.890	1.000	1.000	1.000	1.000	1.000	0.000	0.000	0.000	0.000	0.000	0.000	0.000	0.000	0.000	0.000	0.000
uncultured_Desulfoglaeba_sp.	0.550	0.570	1.000	0.890	1.000	1.000	1.000	1.000	1.000	0.000	0.000	0.000	0.000	0.000	0.000	0.000	0.000	0.000	0.000	0.000
Desulfosporosinus	0.005	0.018	1.000	4.000	1.000	0.007	1.000	1.000	1.000	0.000	0.000	0.000	0.001	0.000	0.000	0.000	0.000	0.000	0.000	0.000
Xylochloris_irregularis	0.470	0.560	1.000	1.000	1.000	0.470	1.000	1.000	1.000	0.000	0.000	0.000	0.001	0.000	0.000	0.000	0.000	0.000	0.000	0.000
uncultured_beta_proteobacterium	0.470	0.560	1.000	1.000	0.470	1.000	1.000	1.000	1.000	0.000	0.000	0.001	0.000	0.000	0.000	0.000	0.000	0.000	0.000	0.000
uncultured_Acidimicrobiales_bacterium	0.470	0.560	1.000	1.000	1.000	0.470	1.000	1.000	1.000	0.000	0.000	0.000	0.001	0.000	0.000	0.000	0.000	0.000	0.000	0.000
Nitrolancea	0.470	0.560	1.000	1.000	1.000	0.470	1.000	1.000	1.000	0.000	0.000	0.000	0.000	0.001	0.000	0.000	0.000	0.000	0.000	0.000
Humibacter	0.470	0.560	1.000	1.000	1.000	0.470	1.000	1.000	1.000	0.000	0.000	0.000	0.000	0.001	0.000	0.000	0.000	0.000	0.000	0.000
Halocella	0.470	0.560	1.000	1.000	1.000	0.470	1.000	1.000	1.000	0.000	0.000	0.000	0.001	0.000	0.000	0.000	0.000	0.000	0.000	0.000
Filimonas	0.470	0.560	1.000	1.000	1.000	0.470	1.000	1.000	1.000	0.000	0.000	0.000	0.001	0.000	0.000	0.000	0.000	0.000	0.000	0.000
Pasteuria	0.550	0.570	1.000	0.890	0.840	1.000	0.840	1.000	1.000	0.000	0.000	0.000	0.000	0.000	0.000	0.000	0.000	0.000	0.000	0.000
Truepera	0.550	0.570	1.000	0.900	1.000	1.000	1.000	1.000	1.000	0.000	0.000	0.000	0.000	0.000	0.000	0.000	0.000	0.000	0.000	0.000
wastewater_metagenome	0.470	0.560	1.000	1.000	1.000	0.470	1.000	1.000	1.000	0.000	0.000	0.000	0.000	0.001	0.000	0.000	0.000	0.000	0.000	0.000
uncultured_Rubrobacteria_bacterium	0.470	0.560	1.000	1.000	0.470	1.000	1.000	1.000	1.000	0.000	0.000	0.001	0.000	0.000	0.000	0.000	0.000	0.000	0.000	0.000
uncultured_Desulfuromonadaceae_bacterium	0.470	0.560	1.000	1.000	1.000	0.470	1.000	1.000	1.000	0.000	0.000	0.000	0.001	0.000	0.000	0.000	0.000	0.000	0.000	0.000
uncultured_Cystobacterineae_bacterium	0.470	0.560	1.000	1.000	1.000	0.470	1.000	1.000	1.000	0.000	0.000	0.000	0.001	0.000	0.000	0.000	0.000	0.000	0.000	0.000
uncultured_Acidimicrobidae_bacterium	0.470	0.560	1.000	1.000	1.000	0.470	1.000	1.000	1.000	0.000	0.000	0.000	0.001	0.000	0.000	0.000	0.000	0.000	0.000	0.000
Thermobacillus	0.470	0.560	1.000	1.000	1.000	1.000	0.470	1.000	1.000	0.000	0.000	0.000	0.000	0.000	0.001	0.000	0.000	0.000	0.000	0.000
Rhizobacter	0.470	0.560	1.000	1.000	0.470	1.000	1.000	1.000	1.000	0.000	0.000	0.001	0.000	0.000	0.000	0.000	0.000	0.000	0.000	0.000
Pseudenhygromyxa	0.470	0.560	1.000	1.000	1.000	0.470	1.000	1.000	1.000	0.000	0.000	0.000	0.001	0.000	0.000	0.000	0.000	0.000	0.000	0.000
Pedobacter	0.470	0.560	1.000	1.000	1.000	0.470	1.000	1.000	1.000	0.000	0.000	0.000	0.001	0.000	0.000	0.000	0.000	0.000	0.000	0.000
PAUC26f	0.470	0.560	1.000	1.000	0.470	1.000	1.000	1.000	1.000	0.000	0.000	0.001	0.000	0.000	0.000	0.000	0.000	0.000	0.000	0.000
Moheibacter	0.470	0.560	1.000	1.000	1.000	0.470	1.000	1.000	1.000	0.000	0.000	0.000	0.001	0.000	0.000	0.000	0.000	0.000	0.000	0.000

	One-way ANOVA				P values of Tukey’s HSD post hoc					Average relative abundance among plant compartments and fertilizer treatments (C: control, N: fertilized)											
Taxa	P Group	FDR Group	P adjusted Group	F Value Group	BS	Rh	R	Stem	Leaf	Average	BS-C mean	BS-N mean	Rh-C mean	Rh-N mean	R-C mean	R-N mean	Stem-C mean	Stem-N mean	Leaf-C mean	Leaf-N mean
Lautropia	0.470	0.560	1.000	1.000	1.000	0.470	1.000	1.000	1.000	0.000	0.000	0.000	0.001	0.000	0.000	0.000	0.000	0.000	0.000	0.000
I8	0.470	0.560	1.000	1.000	0.470	1.000	1.000	1.000	1.000	0.000	0.000	0.001	0.000	0.000	0.000	0.000	0.000	0.000	0.000	0.000
Geminicoccus	0.470	0.560	1.000	1.000	1.000	0.470	1.000	1.000	1.000	0.000	0.000	0.000	0.001	0.000	0.000	0.000	0.000	0.000	0.000	0.000
FukuN18_freshwater_group	0.470	0.560	1.000	1.000	1.000	0.470	1.000	1.000	1.000	0.000	0.000	0.000	0.001	0.000	0.000	0.000	0.000	0.000	0.000	0.000
Candidatus_Glomeribacter	0.470	0.560	1.000	1.000	1.000	1.000	0.470	1.000	1.000	0.000	0.000	0.000	0.000	0.000	0.001	0.000	0.000	0.000	0.000	0.000
Acidisoma	0.470	0.560	1.000	1.000	1.000	1.000	0.470	1.000	1.000	0.000	0.000	0.000	0.000	0.000	0.000	0.001	0.000	0.000	0.000	0.000
Sulfurifustis	0.550	0.570	1.000	0.890	0.760	0.910	1.000	1.000	1.000	0.000	0.000	0.000	0.000	0.000	0.000	0.000	0.000	0.000	0.000	0.000
Silvanigrella	0.550	0.570	1.000	0.890	1.000	1.000	1.000	1.000	1.000	0.000	0.000	0.000	0.000	0.000	0.000	0.000	0.000	0.000	0.000	0.000
uncultured_haloarchaeon	0.470	0.560	1.000	1.000	1.000	0.470	1.000	1.000	1.000	0.000	0.000	0.000	0.000	0.000	0.000	0.000	0.000	0.000	0.000	0.000
uncultured_compost_bacterium	0.470	0.560	1.000	1.000	1.000	0.470	1.000	1.000	1.000	0.000	0.000	0.000	0.000	0.000	0.000	0.000	0.000	0.000	0.000	0.000
uncultured_bacterium_GKS2174	0.470	0.560	1.000	1.000	1.000	0.470	1.000	1.000	1.000	0.000	0.000	0.000	0.000	0.000	0.000	0.000	0.000	0.000	0.000	0.000
Parvibaculum	0.470	0.560	1.000	1.000	1.000	0.470	1.000	1.000	1.000	0.000	0.000	0.000	0.000	0.000	0.000	0.000	0.000	0.000	0.000	0.000
Parachlamydia	0.470	0.560	1.000	1.000	1.000	1.000	0.470	1.000	1.000	0.000	0.000	0.000	0.000	0.000	0.000	0.000	0.000	0.000	0.000	0.000
Luteimonas	0.470	0.560	1.000	1.000	0.470	1.000	1.000	1.000	1.000	0.000	0.000	0.000	0.000	0.000	0.000	0.000	0.000	0.000	0.000	0.000
Longispora	0.470	0.560	1.000	1.000	1.000	0.470	1.000	1.000	1.000	0.000	0.000	0.000	0.000	0.000	0.000	0.000	0.000	0.000	0.000	0.000
Ktedonobacter	0.470	0.560	1.000	1.000	1.000	1.000	0.470	1.000	1.000	0.000	0.000	0.000	0.000	0.000	0.000	0.000	0.000	0.000	0.000	0.000
IMCC26207	0.470	0.560	1.000	1.000	1.000	1.000	0.470	1.000	1.000	0.000	0.000	0.000	0.000	0.000	0.000	0.000	0.000	0.000	0.000	0.000
Heliimonas	0.470	0.560	1.000	1.000	1.000	0.470	1.000	1.000	1.000	0.000	0.000	0.000	0.000	0.000	0.000	0.000	0.000	0.000	0.000	0.000
Elizabethkingia	0.470	0.560	1.000	1.000	1.000	0.470	1.000	1.000	1.000	0.000	0.000	0.000	0.000	0.000	0.000	0.000	0.000	0.000	0.000	0.000
Calothrix_PCC6303	0.470	0.560	1.000	1.000	1.000	0.470	1.000	1.000	1.000	0.000	0.000	0.000	0.000	0.000	0.000	0.000	0.000	0.000	0.000	0.000
Azovibrio	0.470	0.560	1.000	1.000	1.000	0.470	1.000	1.000	1.000	0.000	0.000	0.000	0.000	0.000	0.000	0.000	0.000	0.000	0.000	0.000
Arenimonas	0.470	0.560	1.000	1.000	1.000	0.470	1.000	1.000	1.000	0.000	0.000	0.000	0.000	0.000	0.000	0.000	0.000	0.000	0.000	0.000
Alicyclobacillus	0.470	0.560	1.000	1.000	1.000	0.470	1.000	1.000	1.000	0.000	0.000	0.000	0.000	0.000	0.000	0.000	0.000	0.000	0.000	0.000
Flexibacter	0.540	0.570	1.000	0.900	1.000	1.000	1.000	1.000	1.000	0.000	0.000	0.000	0.000	0.000	0.000	0.000	0.000	0.000	0.000	0.000
Candidatus_Lloydbacteria_bacterium_RIFOXYC12_FULL_46_25	0.540	0.570	1.000	0.900	1.000	1.000	1.000	1.000	1.000	0.000	0.000	0.000	0.000	0.000	0.000	0.000	0.000	0.000	0.000	0.000
uncultured_Crater_Lake_bacterium_CL5004	0.470	0.560	1.000	1.000	1.000	0.470	1.000	1.000	1.000	0.000	0.000	0.000	0.000	0.000	0.000	0.000	0.000	0.000	0.000	0.000
uncultivated_soil_bacterium_clone_C112	0.470	0.560	1.000	1.000	1.000	0.470	1.000	1.000	1.000	0.000	0.000	0.000	0.000	0.000	0.000	0.000	0.000	0.000	0.000	0.000
Turneriella	0.470	0.560	1.000	1.000	1.000	0.470	1.000	1.000	1.000	0.000	0.000	0.000	0.000	0.000	0.000	0.000	0.000	0.000	0.000	0.000
Pseudoneochloris_marina	0.470	0.560	1.000	1.000	1.000	0.470	1.000	1.000	1.000	0.000	0.000	0.000	0.000	0.000	0.000	0.000	0.000	0.000	0.000	0.000
Procabacter	0.470	0.560	1.000	1.000	1.000	1.000	0.470	1.000	1.000	0.000	0.000	0.000	0.000	0.000	0.000	0.000	0.000	0.000	0.000	0.000

	One-way ANOVA				P values of Tukey’s HSD post hoc					Average relative abundance among plant compartments and fertilizer treatments (C: control, N: fertilized)										
Taxa	P Group	FDR Group	P adjusted Group	F Value Group	BS	Rh	R	Stem	Leaf	Average	BS-C mean	BS-N mean	Rh-C mean	Rh-N mean	R-C mean	R-N mean	Stem-C mean	Stem-N mean	Leaf-C mean	Leaf-N mean
Longimicrobium	0.470	0.560	1.000	1.000	1.000	0.470	1.000	1.000	1.000	0.000	0.000	0.000	0.000	0.000	0.000	0.000	0.000	0.000	0.000	0.000
Leptolyngbya_ANT.L52.2	0.470	0.560	1.000	1.000	1.000	0.470	1.000	1.000	1.000	0.000	0.000	0.000	0.000	0.000	0.000	0.000	0.000	0.000	0.000	0.000
JCM_18997	0.470	0.560	1.000	1.000	1.000	0.470	1.000	1.000	1.000	0.000	0.000	0.000	0.000	0.000	0.000	0.000	0.000	0.000	0.000	0.000
Erysipelatoclostridium	0.470	0.560	1.000	1.000	1.000	0.470	1.000	1.000	1.000	0.000	0.000	0.000	0.000	0.000	0.000	0.000	0.000	0.000	0.000	0.000
Chlorophyta_symbiont_of_Lubomirskia_sp.	0.470	0.560	1.000	1.000	1.000	0.470	1.000	1.000	1.000	0.000	0.000	0.000	0.000	0.000	0.000	0.000	0.000	0.000	0.000	0.000
Chloronema	0.470	0.560	1.000	1.000	1.000	0.470	1.000	1.000	1.000	0.000	0.000	0.000	0.000	0.000	0.000	0.000	0.000	0.000	0.000	0.000
Chloromonas_perforata	0.470	0.560	1.000	1.000	1.000	0.470	1.000	1.000	1.000	0.000	0.000	0.000	0.000	0.000	0.000	0.000	0.000	0.000	0.000	0.000
Candidatus_Staskawiczbacteria_bacterium_RIFCSPHIGHO2_01_FULL_34_27	0.470	0.560	1.000	1.000	1.000	0.470	1.000	1.000	1.000	0.000	0.000	0.000	0.000	0.000	0.000	0.000	0.000	0.000	0.000	0.000
Candidatus_Nucleicultrix	0.470	0.560	1.000	1.000	1.000	1.000	0.470	1.000	1.000	0.000	0.000	0.000	0.000	0.000	0.000	0.000	0.000	0.000	0.000	0.000
Anaerobacterium	0.470	0.560	1.000	1.000	1.000	1.000	0.470	1.000	1.000	0.000	0.000	0.000	0.000	0.000	0.000	0.000	0.000	0.000	0.000	0.000
actinobacterium_YJF130	0.470	0.560	1.000	1.000	1.000	0.470	1.000	1.000	1.000	0.000	0.000	0.000	0.000	0.000	0.000	0.000	0.000	0.000	0.000	0.000
Candidatus_Blackburnbacteria_bacterium_RIFCSPLOWO2_01_FULL_44_43	0.550	0.570	1.000	0.890	1.000	1.000	1.000	1.000	1.000	0.000	0.000	0.000	0.000	0.000	0.000	0.000	0.000	0.000	0.000	0.000
Zavarzinella	0.470	0.560	1.000	1.000	1.000	1.000	0.470	1.000	1.000	0.000	0.000	0.000	0.000	0.000	0.000	0.000	0.000	0.000	0.000	0.000
uncultured_prokaryote	0.470	0.560	1.000	1.000	1.000	0.470	1.000	1.000	1.000	0.000	0.000	0.000	0.000	0.000	0.000	0.000	0.000	0.000	0.000	0.000
Spirochaeta_2	0.470	0.560	1.000	1.000	1.000	0.470	1.000	1.000	1.000	0.000	0.000	0.000	0.000	0.000	0.000	0.000	0.000	0.000	0.000	0.000
CL5003	0.470	0.560	1.000	1.000	1.000	0.470	1.000	1.000	1.000	0.000	0.000	0.000	0.000	0.000	0.000	0.000	0.000	0.000	0.000	0.000
Blastopirellula	0.470	0.560	1.000	1.000	1.000	0.470	1.000	1.000	1.000	0.000	0.000	0.000	0.000	0.000	0.000	0.000	0.000	0.000	0.000	0.000
Alcaligenes	0.470	0.560	1.000	1.000	1.000	0.470	1.000	1.000	1.000	0.000	0.000	0.000	0.000	0.000	0.000	0.000	0.000	0.000	0.000	0.000
uncultured_Parcubacteria_group_bacterium	0.470	0.560	1.000	1.000	1.000	0.470	1.000	1.000	1.000	0.000	0.000	0.000	0.000	0.000	0.000	0.000	0.000	0.000	0.000	0.000
uncultured_Desulfocaldus_sp.	0.470	0.560	1.000	1.000	1.000	0.470	1.000	1.000	1.000	0.000	0.000	0.000	0.000	0.000	0.000	0.000	0.000	0.000	0.000	0.000
Turicibacter	0.470	0.560	1.000	1.000	1.000	0.470	1.000	1.000	1.000	0.000	0.000	0.000	0.000	0.000	0.000	0.000	0.000	0.000	0.000	0.000
Tistrella	0.470	0.560	1.000	1.000	1.000	0.470	1.000	1.000	1.000	0.000	0.000	0.000	0.000	0.000	0.000	0.000	0.000	0.000	0.000	0.000
Ignavibacterium	0.470	0.560	1.000	1.000	1.000	0.470	1.000	1.000	1.000	0.000	0.000	0.000	0.000	0.000	0.000	0.000	0.000	0.000	0.000	0.000

Table S4. Relative abundances (% of total good-quality sequences) of top 30 genera (> 0.22% average relative abundance) in each compartment of water yam (*Dioscorea alata* L.) cv. A-19 cultivated with different levels of fertilizers. BS-C, Bulk soil on control; BS-N, Bulk soil on nitrogen treatment; Rh-C, Rhizosphere on control; Rh-N, Rhizosphere on nitrogen treatment; R-C, Root on control; R-N, Root on nitrogen treatment; Stem-C, Stem on control; Stem-N, Stem on nitrogen treatment; Leaf-C, Leaf on control; Leaf-N, Leaf on nitrogen treatment. +: significantly different between Control and Treatment using Tukey's HSD multiple comparison test.

Phylum	Class	Order	Familly	Genus	Average	BS-C	BS-N	p <0.05	Rh-C	Rh-N	p <0.05	R-C	R-N	p <0.05	Stem_C	Stem_N	p <0.05	Leaf_C	Leaf_N	p <0.05
				Unclassified	34.14	39.56	42.51		44.76	40.83		25.30	29.16		31.47	41.86		29.70	16.24	
Proteobacteria	*Alphaproteobacteria*	*Rhizobiales*	*Rhizobiaceae*	*AllorhizobiumNeorhizobium PararhizobiumRhizobium*	9.40	6.76	1.96		8.88	8.35		21.16	12.25		6.30	6.86		16.89	4.62	
Proteobacteria	*Betaproteobacteria*	*Burkholderiales*	*Burkholderiaceae*	*BurkholderiaCaballeroniaP araburkholderia*	8.15	0.04	0.23		6.76	19.45		20.98	21.90		12.04	0.00	+	0.04	0.09	
Proteobacteria	*Gammaproteobacteria*	*Xanthomonadales*	*Xanthomonadaceae*	*Stenotrophomonas*	4.67	0.00	0.00		0.09	0.44		0.98	4.20		3.03	3.42		12.53	22.00	
Proteobacteria	*Gammaproteobacteria*	*Pseudomonadales*	*Pseudomonadaceae*	*Pseudomonas*	4.56	2.02	0.94		0.02	0.01		0.26	0.06		9.12	16.40		4.71	12.11	
				uncultured_bacterium	2.70	15.68	9.12		1.42	0.36		0.03	0.00		0.01	0.03		0.30	0.00	
				uncultured	1.94	6.50	8.88		2.62	0.90		0.29	0.12		0.07	0.05		0.00	0.00	
Actinobacteria	*Actinobacteria*	*Glycomycetales*	*Glycomycetaceae*	*Glycomyces*	1.01	0.00	0.00		0.10	0.58		0.90	7.90	+	0.00	0.02		0.59	0.00	
Proteobacteria	*Betaproteobacteria*	*Burkholderiales*	*Burkholderiaceae*	*Ralstonia*	1.01	0.08	0.01		1.59	4.49		2.10	1.44		0.18	0.10		0.06	0.01	
Actinobacteria	*Actinobacteria*	*Streptomycetales*	*Streptomycetaceae*	*Streptomyces*	1.01	0.00	0.07		2.22	2.28		2.86	1.69		0.00	0.49		0.46	0.00	
Firmcutes	*Bacilli*	*Bacillales*	*Bacillaceae*	*Anaerobacillus*	0.79	3.61	0.76		0.00	0.00		0.00	0.00		0.12	0.15		3.24	0.06	
Bacteroidetes	*Sphingobacteriia*	*Sphingobacteriales*	*Sphingobacteriaceae*	*Olivibacter*	0.70	0.00	0.00		0.13	0.62		0.06	1.25		1.19	0.83		0.44	2.46	
Proteobacteria	*Betaproteobacteria*	*Burkholderiales*	*Comamonadaceae*	*Delftia*	0.62	2.10	4.00		0.00	0.00		0.01	0.00		0.17	0.31		3.03	0.03	+
Proteobacteria	*Alphaproteobacteria*	*Rhizobiales*	*Bradyrhizobiaceae*	*Bradyrhizobium*	0.61	0.14	0.17		1.25	2.28		1.17	0.41		0.04	0.35		0.10	0.15	
Proteobacteria	*Gammaproteobacteria*	*Xanthomonadales*	*Rhodanobacteraceae*	*Dyella*	0.56	0.00	0.04		1.66	0.92		2.43	0.50	+	0.01	0.00		0.00	0.00	
Proteobacteria	*Betaproteobacteria*	*Burkholderiales*	*Burkholderiaceae*	*Cupriavidus*	0.50	0.01	0.06		1.61	1.90		1.00	0.45		0.00	0.00		0.00	0.00	
Proteobacteria	*Alphaproteobacteria*	*Rhizobiales*	*Xanthobacteraceae*	*Labrys*	0.44	0.00	0.00		0.22	0.36		1.21	1.17		0.30	0.42		0.21	0.50	
Proteobacteria	*Gammaproteobacteria*	*Enterobacterales*	*Enterobacteriaceae*	*Enterobacter*	0.42	0.00	0.00		0.00	0.02		0.00	0.04		3.96	0.18		0.00	0.00	
Firmcutes	*Bacilli*	*Bacillales*	*Staphylococcaceae*	*Staphylococcus*	0.42	2.72	0.05	+	0.03	0.01		0.00	0.00		0.08	0.48		0.71	0.09	
Proteobacteria	*Betaproteobacteria*	*Burkholderiales*	*Alcaligenaceae*	*Achromobacter*	0.39	0.00	0.00		0.02	0.14		0.28	2.72	+	0.04	0.08		0.45	0.15	
Firmcutes	*Bacilli*	*Bacillales*	*Paenibacillaceae*	*Paenibacillus*	0.33	0.02	0.04		0.12	0.07		2.16	0.77		0.03	0.00		0.12	0.00	
Firmcutes	*Bacilli*	*Bacillales*	*Bacillaceae*	*Bacillus*	0.33	2.02	0.49		0.07	0.04		0.04	0.02		0.20	0.37		0.00	0.05	
Actinobacteria	*Rubrobacteria*	*Gaiellales*	*Gaiellaceae*	*Gaiella*	0.28	1.61	1.08		0.10	0.02		0.00	0.00		0.00	0.00		0.00	0.00	
Proteobacteria	*Alphaproteobacteria*	*Sphingomonadales*	*Sphingomonadaceae*	*Sphingobium*	0.27	0.00	0.04		1.35	0.55		0.58	0.17		0.00	0.00		0.00	0.00	
Bacteroidetes	*Flavobacteriia*	*Flavobacteriales*	*Weeksellaceae*	*Chryseobacterium*	0.26	0.00	0.00		0.17	0.40		0.01	0.07		0.62	0.29		0.00	1.00	
Proteobacteria	*Alphaproteobacteria*	*Rhodospirillales*	*Rhodospirillaceae*	*Inquilinus*	0.25	0.01	0.00		0.48	0.38		0.88	0.69		0.03	0.00		0.00	0.00	
Proteobacteria	*Alphaproteobacteria*	*Rhizobiales*	*Xanthobacteraceae*	*Pseudolabrys*	0.25	0.22	0.49		0.35	0.38		0.23	0.26		0.08	0.34		0.03	0.08	
Actinobacteria	*Actinobacteria*	*Propionibacteriales*	*Nocardioidaceae*	*Kribbella*	0.22	0.00	0.14		0.64	1.39		0.01	0.04		0.00	0.00		0.00	0.00	
Bacteroidetes	*Chitinophagia*	*Chitinophagales*	*Chitinophagaceae*	*Niastella*	0.22	0.01	0.18	+	1.42	0.55	+	0.00	0.00		0.00	0.00		0.00	0.00	
Firmcutes	*Bacilli*	*Lactobacillales*	*Streptococcaceae*	*Lactococcus*	0.22	0.32	0.00		0.00	0.00		0.00	0.00		1.72	0.03		0.08	0.00	

>20%
>10%
>5%
>1%

Table S5 Comparison of PGPB associated with water yam (*Dioscorea alata* L.) by cultural dependent methods in our previous study and by amplicon profiling in the present study.

Isolated bacteria					Ability		Genera and clades identifed on amplicon sequence
Genus	Accession	Phylum	Class	Isolated by	Possible Function	Reference	
Azospirillum sp.	A-19, E-2	*Proteobacteria*	*Alphaproteobacteria*	Rezaei *et al*., 2017; Takada *et al.,* 2019	Nitrogen fixation	Araújo et al., 2013; Rodorigues et al., 2015	*Azospirillum*
Bradyrhizobium sp.	E-1	*Proteobacteria*	*Alphaproteobacteria*	Ouyabe *et al*., 2019a	Nitrogen fixation, IAA production, ACC deaminase activity	Terakado-Tonooka et al.,2008, 2013 Hara et al., 2019;Ikeda et al 2014	*Bradyrhizobium*
Devosia sp.	A-19,A-58, A-62, A-133 E-2	*Proteobacteria*	*Alphaproteobacteria*	Rezaei *et al*., 2017, Ouyabe *et al*., 2019a, 2019b, Shiwachi *et al*., 2020	Nitrogen fixation	Rivas et al., 2002	*Devosia*
Labrys sp.	A-19	*Proteobacteria*	*Alphaproteobacteria*	Shiwachi *et al*., 2020	Nitorgen fixation, IAA production, Phosphate solubliization ,Siderophore production	Valdez-Nuñez et al., 2019	*Labrys*
Neorhizobium sp.	A-19,E-3	*Proteobacteria*	*Alphaproteobacteria*	Ouyabe *et al*., 2019a, 2019b	Nitrogen fixation	Ouyabe et al., 2019a,	*AllorhizobiumNeorhizobium PararhizobiumRhizobium*
Rhizobium sp.	A-18, A-19, A-23, A-61, A-62, A-112,A-113, A-116 , E-1, E-2,E-3	*Proteobacteria*	*Alphaproteobacteria*	Rezaei *et al*., 2017; Takada *et al*., 2019; Ouyabe *et al*., 2019a, 2019b, 2019c; Shiwachi *et al* 2020	Nitrogen fixation, IAA production, ACC deaminase activity	Duan et al.,2009, Habibi et al., 2019. Nuñez et al., 2019	
Rhizobium dioscoreae sp.	A-62,E-1	*Proteobacteria*	*Alphaproteobacteria*	Ouyabe *et al*., 2020a	Nitrogen fixation, IAA production, Siderophore production, Phosphates solublization	Ouyabe *et al*., 2020	
Stenotrophomonas sp.	A-19,A-133, E-3	*Proteobacteria*	*Alphaproteobacteria*	Ouyabe *et al*., 2019b, 2019c; Shiwachi et al., 2020	Nitrogen fixation, IAA production, Siderophore production, Phosphates solublization	Taulé et al., 2012	*Stenotrophomonas*
Achromobacter sp.	A-18, A-68	*Proteobacteria*	*Betaproteobacteria*	Ouyabe *et al*., 2019a, 2019b	Nitrogen fixation, IAA production, Phosphate solublization	Taulé et al., 2012	*Achromobacter*
Burkholderia sp.	A-112,A-18,E-1,E-3	*Proteobacteria*	*Betaproteobacteria*	Ouyabe *et al.* , 2019a,2019b,2019c	IAA production, Nitogen fixation, ACC deaminase activity	Knief et al., 2012;Araújo et al., 2013, Terakado-Tonooka et al.,2008,2013 Ikeda et al., 2014;Souza et al 2015	*BurkholderiaCaballeronia Paraburkholderia*
Paraburkholderia sp.	A-133,E-2	*Proteobacteria*	*Betaproteobacteria*	Ouyabe *et al*., 2019b,2019c	Nitrogen fixation, IAA ,and Siderophore production, Phosphates solublization ACC deaminase activity	Kuramae et al., 2020; Herpell et al. 2020	
Ralstonia sp.	A-19,A-133	*Proteobacteria*	*Betaproteobacteria*	Takada *et al*., 2019; Ouyabe *et al.,* 2019b	Nitrogen fixation	Chen et al., 2001	*Ralstonia*
Acinetobacter sp.	E-1,E-2,E-3	*Proteobacteria*	*Gammaproteobacteria*	Ouyabe *et al*., 2019c	Nitrogen fixation, IAA production, Siderophore Production, Phosphates solubilizaiton	Taulé et al., 2012	*Acinetobacter*
Enterobacter sp.	A-17, A-19,A-133, E-1, E-2, E-3	*Proteobacteria*	*Gammaproteobacteria*	Takada *et al*., 2019; Ouyabe *et al.,* 2019a, 2019b, 2019c	Nitrogen fixation, IAA and, Siderophore Production, Phosphates solubilizaiton	Asis and Adachi,2004 Taulé et al., 2012	*Enterobacter*
Lysobacter sp.	A-18	*Proteobacteria*	*Gammaproteobacteria*	Ouyabe *et al*., 2019b	Nitrogen fixation, Anti-microbial compound production,	Expósito et al 2015;	*Lysobacter*
Pseudoxanthomonas sp.	A-68	*Proteobacteria*	*Gammaproteobacteria*	Ouyabe *et al*., 2019a	IAA and other auxin production, Phosphate Solublization,	Marasco et al., 2013, Souza et al., 2015	*Pseudoxanthomonas*
Pseudomonas sp.	A-133,E-1,E-3	*Proteobacteria*	*Gammaproteobacteria*	Ouyabe *et al.* , 2019b,2019c	Nitrogen fixation, IAA Production, Siderophore production, Phosphates solublization	Taulé et al., 2012	*Pseudomonas*
Xanthomonas sp.	A-17, A-133, E-1, E-2, E-3	*Proteobacteria*	*Gammaproteobacteria*	Rezaei *et al*., 2017; Ouyabe *et al*.,2019a, 2019b, 2019c	Nitrogen fixation, Siderophore solubilization	Taulé et al., 2012	*Xanthomonas*
Bacillus sp.	A-4, A-18, A-19,A-44, A-61, A-62, A-73,A-86 E-2, E-3	*Firmicutes*	*Bacilli*	Takada *et al*., 2019; Ouyabe *et al*., 2019a, 2019b,2019c; Shiwachi *et al*., 2020	Nitrogen fixation, IAA production Siderophore production,	Magnani et al., 2010; Ji et al., 2014, Souza e al., 2015	*Bacillus*
Paenibacillus sp.	A-18, A-19,A-23,A-133, E-2	*Firmcutes*	*Bacilli*	Ouyabe *et al*., 2019a,2019b, 2019c; Shiwachi *et al*., 2020	Nitrogen fixation, IAA and other auxin production, Phosphate solublization, ACC deaminase activity	Xie et al. 2014; Souza et al.,2015; Grady et al.,2016	*Paenibacillus*
Staphylococcus sp.	A-19	*Firmicutes*	*Bacilli*	Ouyabe *et al*., 2019b	Nitrogen fixation, IAA production, Phosphate solublization ACC deaminase activity	Shahid et al., 2019; Araújo et al., 2020	*Staphylococcus*
Mycobacterium sp.	A-86	*Actinobacteria*	*Actinobacteria*	Ouyabe *et al*., 2019a	Nitrogen fixation Phosphate solublization. ACC deaminase activity	Sathya et al., 2017 Valdez-Nuñez et al., 2019	*Mycobacterium*
Microbacterium sp.	A-19, A-62, , A-133,E-2, E-3	*Actinobacteria*	*Actinobacteria*	Ouyabe *et al*., 2019b,2019c; Shiwachi *et al*., 2020	Nitrogen fixation	Lin et al., 2012	*Microbacterium*
Streptomyces sp.	A-19,E-3	*Actinobacteria*	*Actinobacteria*	Ouyabe *et al*., 2019b,2019c	Nitrogen fixation, Phosphate solublization, Siderophore production IAA production, ACC deaminase activity	Sathya et al., 2017	*Streptomyces*

Asis, C. A., and Adachi, K. (2004). Isolation of endophytic diazotroph Pantoea agglomerans and nondiazotroph Enterobacter asburiae from sweetpotato stem in Japan. *Lett Appl Microbiol* 38, 19–23.

Araújo, A. E. da S., Baldani, V. L. D., Galisa, P. de S., Pereira, J. A., and Baldani, J. I. (2013). Response of traditional upland rice varieties to inoculation with selected diazotrophic bacteria isolated from rice cropped at the Northeast region of Brazil. Appl Soil Ecol 64, 49–55.

Chen, W. M., S. Laevens, T. M. Lee, T. Coenye, P. D. Vos, M. Mergeay, and P. Vandamme 2001. Ralstonia taiwanensis sp. nov., isolated from root nodules of Mimosa species and sputum of a cystic fibrosis patient. Int. J. Syst. Evol. Microbiol. 51

Duan, J., Müller, K. M., Charles, T. C., Vesely, S., and Glick, B. R. (2009). 1-Aminocyclopropane-1-Carboxylate (ACC) Deaminase Genes in Rhizobia from Southern Saskatchewan. Microbial Ecol 57, 423–436.

Expósito, R. G., Postma, J., Raaijmakers, J. M., and Bruijn, I. D. (2015). Diversity and Activity of Lysobacter Species from Disease Suppressive Soils. *Front Microbiol* 6, 1243.

Grady, E. N., MacDonald, J., Liu, L., Richman, A., and Yuan, Z.-C. (2016). Current knowledge and perspectives of Paenibacillus: a review. *Microb Cell Fact* 15, 203.

Habibi, S., Djedidi, S., Ohkama-Ohtsu, N., Sarhadi, W. A., Kojima, K., Rallos, R. V., et al. (2019). Isolation and Screening of Indigenous Plant Growth-promoting Rhizobacteria from Different Rice Cultivars in Afghanistan Soils. *Microbes Environment* 34, 347–355.

Hara, S., Morikawa, T., Wasai, S., Kasahara, Y., Koshiba, T., Yamazaki, K., et al. (2019). Identification of Nitrogen-Fixing Bradyrhizobium Associated With Roots of Field-Grown Sorghum by Metagenome and Proteome Analyses. Front Microbiol 10, 407.

Ji, S. H., Gururani, M. A., and Chun, S.-C. (2014). Isolation and characterization of plant growth promoting endophytic diazotrophic bacteria from Korean rice cultivars. *Microbiol Res* 169, 83–98.

Kuramae, E. E., Derksen, S., Schlemper, T. R., Dimitrov, M. R., Costa, O. Y. A., and Silveira, A. P. D. da (2020). Sorghum Growth Promotion by Paraburkholderia tropica and Herbaspirillum frisingense: Putative Mechanisms Revealed by Genomics and Metagenomics. *Microorg* 8, 725.

Lin, L., Guo, W., Xing, Y., Zhang, X., Li, Z., Hu, C., et al. (2012). The actinobacterium Microbacterium sp. 16SH accepts pBBR1-based pPROBE vectors, forms biofilms, invades roots, and fixes N2 associated with micropropagated sugarcane plants. *Appl Microbiol Biot* 93, 1185–1195.

Magnani, G. S., Didonet, C. M., Cruz, L. M., Picheth, C. F., Pedrosa, F. O., and Souza, E. M. (2010). Diversity of endophytic bacteria in Brazilian sugarcane. *Genet Mol Res* 9, 250–258.

Marasco, R., Rolli, E., Fusi, M., Cherif, A., Abou-Hadid, A., El-Bahairy, U., et al. (2013). Plant Growth Promotion Potential Is Equally Represented in Diverse Grapevine Root-Associated Bacterial Communities from Different Biopedoclimatic Environments. *Biomed Res Int* 2013, 1–17.

Rivas, R., E. Velazquez, A. Willems, N. Vizcaíno, N. S. Subba-Rao, P. F. Mateos, M. Gillis, F. B. Dazzo, and E. Martínez-Molina 2002. A new species of Devosia that forms a unique nitrogenfixing root-nodule symbiosis with the aquatic legume Neptunia natans (L.f.) Druce. Applied and environmental microbiology 68: 5217-5222.

Rodrigues, A. C., A. Bonifacio, F. F. de Araujo, M. A. L. Junior, and M. do V. B. Figueiredo 2015. Azospirillum sp. as a Challenge for Agriculture. D.K. Maheshwari (ed.), Bacterial Metabolites in Sustainable Agroecosystem, Sustainable Development and Biodiversity 12: 29-51.

Shahid, M., Ahmed, T., Noman, M., Javed, M. T., Javed, M. R., Tahir, M., et al. (2019). Non-pathogenic Staphylococcus strains augmented the maize growth through oxidative stress management and nutrient supply under induced salt stress. *Ann Microbiol* 69, 727–739.

Souza, R. de, Meyer, J., Schoenfeld, R., Costa, P. B. da, and Passaglia, L. M. P. (2015). Characterization of plant growth-promoting bacteria associated with rice cropped in iron-stressed soils. *Ann Microbiol* 65, 951–964.

Valdez-Nuñez, R. A., Castro-Tuanama, R., Castellano-Hinojosa, A., Bedmar, E. J., and Ríos-Ruiz, W. F. (2019). Microbial Probiotics for Agricultural Systems, Advances in Agronomic Use. *Sustain Plant Crop Prot* , 111–126.

Xie, J.-B., Du, Z., Bai, L., Tian, C., Zhang, Y., Xie, J.-Y., et al. (2014). Comparative Genomic Analysis of N2-Fixing and Non-N2-Fixing Paenibacillus spp.: Organization, Evolution and Expression of the Nitrogen Fixation Genes. *Plos Genet* 10, e1004231.

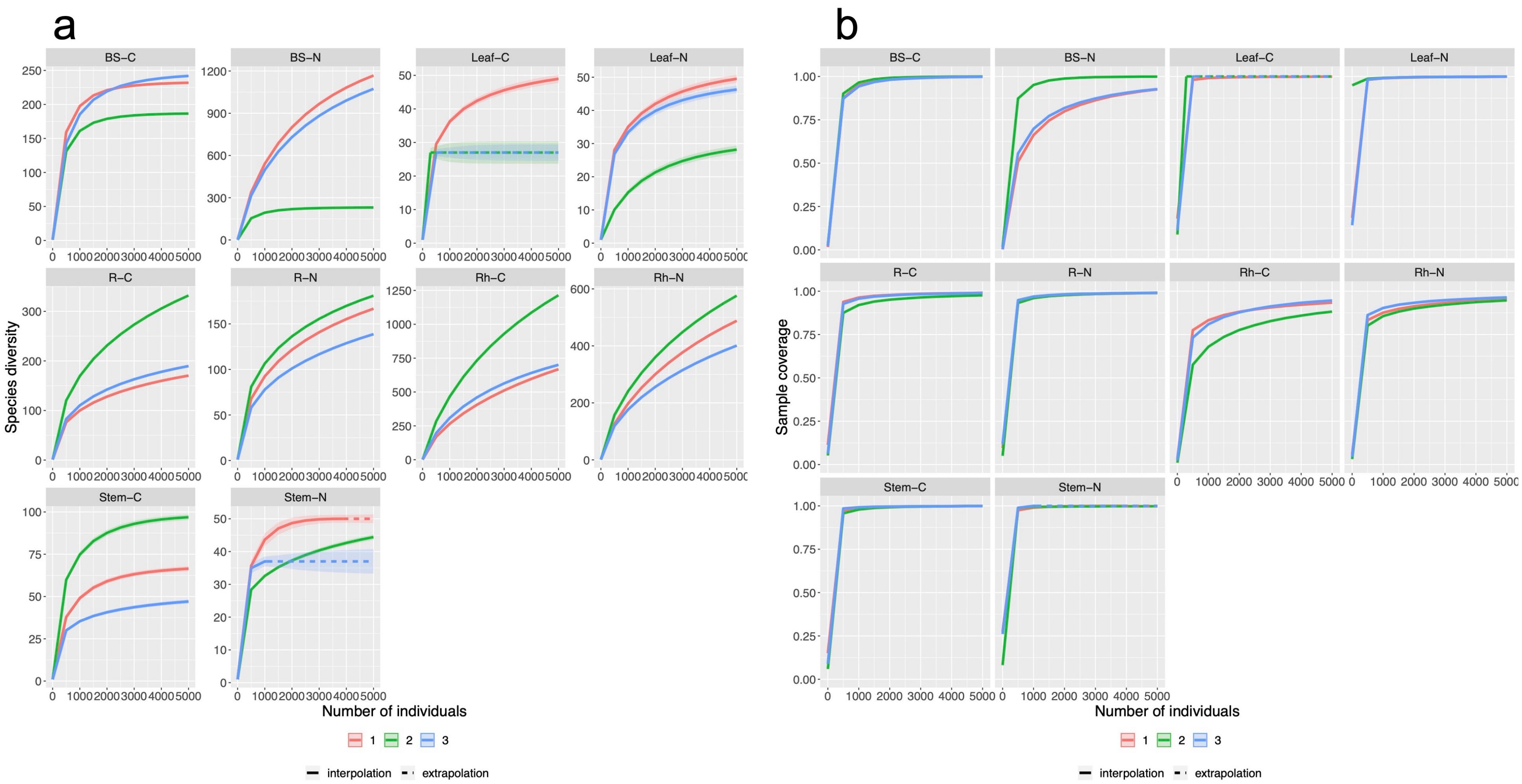

Fig. S1a, b. Sample-size-based Rarefaction curve (a) and Sample completeness curve (b), calculated on interpolation (solid line segment) and extrapolation (dotted line segments) of each plant compartment of water yam cv.A-19. Both curves were calculated using species diversity with 50 bootstrap replicates per sample by iNEXT package in R. The three replicates of each plant compartment are shown in red, green, and blue.
